# Evaluation of the Uncertainty in Satellite-Based Crop State Variable Retrievals Due to Site and Growth Stage Specific Factors and Their Potential in Coupling with Crop Growth Models

**DOI:** 10.3390/rs11161928

**Published:** 2019-08-02

**Authors:** Nathaniel Levitan, Yanghui Kang, Mutlu Özdoğan, Vincenzo Magliulo, Paulo Castillo, Fred Moshary, Barry Gross

**Affiliations:** 1 Department of Electrical Engineering, City College of New York, 160 Convent Ave., New York, NY 10031, USA; 2 Department of Geography, University of Wisconsin-Madison, 550 N. Park St., Madison, WI 53706, USA; 3 Nelson Institute Center for Sustainability and the Global Environment, University of Wisconsin-Madison, 1710 University Avenue, Madison, WI 53726, USA; 4 Department of Forest and Wildlife Ecology, University of Wisconsin-Madison, 1630 Linden Drive, Madison, WI 53706, USA; 5 CNR-Institute of Mediterranean Forest and Agricultural Systems, 85 Via Patacca, 80040-Ercolano (Napoli), Italy; 6 Department of Electrical and Computer Engineering Technology, Farmingdale State College, 2350 Broadhollow Road, Farmingdale, NY 11735-1021, USA

**Keywords:** LAI, GPP, MODIS, LANDSAT, G × E × M, crop growth models, CO_2_ flux towers, Ameriflux, GHG-Europe

## Abstract

Coupling crop growth models and remote sensing provides the potential to improve our understanding of the genotype x environment x management (G × E × M) variability of crop growth on a global scale. Unfortunately, the uncertainty in the relationship between the satellite measurements and the crop state variables across different sites and growth stages makes it difficult to perform the coupling. In this study, we evaluate the effects of this uncertainty with MODIS data at the Mead, Nebraska Ameriflux sites (US-Ne1, US-Ne2, and US-Ne3) and accurate, collocated Hybrid-Maize (HM) simulations of leaf area index (LAI) and canopy light use efficiency (LUE_Canopy_). The simulations are used to both explore the sensitivity of the satellite-estimated genotype × management (G × M) parameters to the satellite retrieval regression coefficients and to quantify the amount of uncertainty attributable to site and growth stage specific factors. Additional ground-truth datasets of LAI and LUE_Canopy_ are used to validate the analysis. The results show that uncertainty in the LAI/satellite measurement regression coefficients lead to large uncertainty in the G × M parameters retrievable from satellites. In addition to traditional leave-one-site-out regression analysis, the regression coefficient uncertainty is assessed by evaluating the retrieval performance of the temporal change in LAI and LUE_Canopy_. The weekly change in LAI is shown to be retrievable with a correlation coefficient absolute value (|r|) of 0.70 and root-mean square error (RMSE) value of 0.4, which is significantly better than the performance expected if the uncertainty was caused by random error rather than secondary effects caused by site and growth stage specific factors (an expected |r| value of 0.36 and RMSE value of 1.46 assuming random error). As a result, this study highlights the importance of accounting for site and growth stage specific factors in remote sensing retrievals for future work developing methods coupling remote sensing with crop growth models.

## 1. Introduction

### 1.1. Background

Mechanistic crop growth models temporally predict the growth of crops as a function of genotype x environment x management (G × E × M) factors [1]. By mechanistically modeling the effects of G × E × M factors and their interactions, crop growth models are able to integrate information about the properties of the seed (genotype), the decisions farmers make both at planting and within the season (management), and the variability in the weather and soil (environment). Examples of these factors in each category of G × E × M are shown in Table 1 [2,3]. In addition to these G × E × M factors, biotic stresses—such as weeds, pests, and diseases—can further limit the growth of crops and these factors are difficult to model, although some recent advances have been made [4]. Nevertheless, in highly developed cropping systems, such as the US corn belt, fields tend to be well-managed and the reduction in yield caused by unmodeled factors, such as biotic stresses, is generally 20% or less [5,6]. As a result, mechanistic crop growth model simulations are able to provide valuable information with relatively strong predictive performance in highly developed cropping systems [6,7].

Assimilation of remote sensing data into crop growth models can be used to reduce the uncertainty in the G × E × M factors (which control crop growth) via calibration [8–11]. In the calibration approach to remote sensing data assimilation, the model parameters and G × E × M factors affecting crop growth are adjusted by reinitialization until the crop growth model output agrees with the remote sensing observation (as opposed to the updating or forcing approaches where the crop model state variables are themselves directly altered) [9]. However, uncertainty in the remote sensing retrievals of crop state variables, such as leaf area index (LAI), leads to significant challenges [9] in the calibration and determination of the G × E × M factors. This is because the interactions of G × E × M factors in crop growth models are highly non-linear and careful application of inversion techniques is required to determine input parameters from observations [12,13]. As a result, even small uncertainties in the remote sensing retrievals can propagate into significant errors in the G × E × M factors determined by calibration [14]. Therefore, calibration of crop models with remote sensing data is primarily used to analyze output variables, such as yields and biomass, discarding the G × E × M factors determined by calibration as an intermediate step [8,15–18].

Nevertheless, improved understanding of the G × E × M factor variability can greatly improve our ability to use crop growth models at the regional scale [6,19,20] to predict into the future and answer questions about climate change [21], agricultural policies [22,23], and yield gaps [24]. At the regional scale, G × E × M parameter uncertainty is even more significant due to a lack of calibration data as compared to the field-scale [1,25]. Thus, constraints from measurements other than yield are vital for further reduction in the uncertainty [25] at this scale. Illustrating this point, ref. [25] found that the majority of the uncertainty in LAI simulations for regional simulations of Indian groundnut was parametric uncertainty, indicating the potential of reductions in the uncertainties of satellite retrievals (such as those of LAI) to significantly improve our understanding of G × E × M variability in calibration of regional crop models [26].

The crop state variable retrieval uncertainty is in a large part caused by the variability in secondary factors [27–32] that influence the remote sensing measurements, such as cultivar type, soil background, canopy structure, and inherent leaf properties; most of these secondary factors are strongly dependent on site and growth stage [33–36]. Physical canopy radiative transfer models, such as PROSAIL [37], provide a theoretical model to understand the effect of the secondary factors by forward modeling the top-of-canopy reflectance spectrum from variables describing the soil background, canopy structure, and leaf properties [9]. However, inversion of canopy radiative transfer models is ill-posed [38] and requires the use of *a priori* constraints to perform the retrievals [39,40]. While temporal [40–42] and spatial [40,43] constraints can be used to address the ill-posedness of the retrieval, they are not sufficiently powerful to remove the uncertainty. As a result, assumptions must be made about the canopy structure and leaf properties [40]. Unfortunately, although both canopy structure and leaf properties have a significant effect on the uncertainty of the retrieval [32], it is difficult to constrain them beyond finding appropriate ranges for the values based on land cover [44] and selecting vegetation indices with greater sensitivity to the variable of interest [32,45,46]. However, even though the full spectral modeling can optimize the best choice of vegetation indices for given applications, using vegetation indices in the retrievals directly still results in valuable spectral information being lost, undercutting the benefits of the possibility of using the full spectral information available with canopy radiative transfer models in the retrieval itself [47] as full-spectrum methods have shown good results in the literature [48,49].

However, because of the lack of information available to remove the uncertainty about secondary factors, physical radiative transfer approaches have not dominated over empirical approaches, although these often do not use the full spectral information available from the sensor and lack a theoretical basis to control secondary factors [27–29]. The empirical algorithms overcome these issues by directly using training data to learn to use the “subtle spectral features to reduce undesired effects” [47] that make vegetation retrievals difficult. In addition, in some cases, empirical methods are also able to improve the retrievals with auxiliary information [29,50,51].

In empirical approaches, the uncertainty caused by the variability in secondary factors manifests as the “one place, one time, one equation” issue [27] where regressions between the satellite measurements and the crop state variables trained on one set of sites and times do not generalize well to another set of sites and times [27,28]. The issue occurs because most empirical studies develop a global regression relating the satellite measurements to the crop state variables which does not account for the spatiotemporal variability in the secondary factors, although some studies have attempted to use the secondary factors to improve the retrieval [29,50,51]. Specifically, refs. [50,51] find that developing separate regression models for different growth stages provides the best results, while [29] finds that including cultivar, planting pattern, and growth stage in the model could improve the performance of the retrievals. While the secondary factors in [29,50,51] do not correspond to the secondary factors in physical radiative transfer models such as PROSAIL, their indirect connection to the leaf and canopy parameters used by PROSAIL [33–36] allows them to reduce the uncertainty caused by the secondary effects. Nevertheless, the work on including secondary effects is quite limited and hampered by lack of available data [28] to span the large spatiotemporal variability in these secondary factors, calling for new approaches to address this issue.

In order to address the uncertainty caused by secondary factors, it is necessary to obtain data that covers the extent of their spatiotemporal variability. Crop growth models provide one possible avenue to obtain information on the secondary factor leaf and soil properties. The use of crop growth models to obtain information about the secondary factors has been best explored in coupling studies [52–55], where remote sensing data is assimilated into a combined model consisting of a crop growth model, a canopy radiative transfer model, and formalisms linking the outputs of the growth model with the inputs of the radiative transfer model. These studies [52–55] have been successful in coupling several variables from the crop growth models, such as LAI, leaf structure parameter, water content, dry matter content, total chlorophyll content, and relative soil dryness. The variables coupled in addition to LAI are secondary factors that affect LAI retrieval [32] and the coupling can be understood to provide constraints on these secondary factors from the biological mechanics of growth and its interaction with the weather/soil environment. In addition, if available, any genetic (cultivar choice) or management information inputted into the crop model can provide additional constraints on the secondary factors [56]. Unfortunately, it is difficult to use crop growth models to gain information about these secondary parameters at a regional scale as information about G × M parameters is limited at this scale [57]. As a result, regional crop growth model simulations are generally validated only against crop yields and phenological dates [6,20,58–60] and consequently may have significant uncertainty in their prediction of in-season state variables (many of which are secondary factors in LAI retrieval) [61]. In contrast, field-scale crop growth model simulations have been validated in much more detail with respect to in-season state variables. For example, several studies [2,62–65] evaluate their performance in predicting LAI, canopy cover, biomass, soil moisture, soil nitrogen, plant nitrogen, evapotranspiration, and phenology as well as yield. The crop model’s stronger performance at field-scale in predicting both the yield and individual within-season process can be attributed to the availability of significantly more accurate agromanagement information, and to a lesser extent to more accurate soil and weather data, at this scale [66]. Thus, incorporating field-scale crop growth modeling of secondary parameters in training and testing agricultural satellite retrieval algorithms [67] can potentially provide for significant advances in addressing the uncertainty caused by site and growth stage specific secondary factors.

### 1.2. Overview

In this study, we seek to show that the difficulties in using remote sensing to determine the G × E × M factors affecting crop growth are strongly connected to variability in the relationship of satellite measurments and crop state variables and that the variability in the relationship is in a large part caused by site and growth stage specific factors. In order to achieve these objectives, this study uses field-scale crop growth model simulations powered by accurate agromanagement information and collocated with satellite data at the Mead, Nebraska Ameriflux sites, supplemented by ground-truth data from additional sites for validation. Crop growth model simulations are used from only the Mead, Nebraska Ameriflux sites because geolocated agromanagement information, vital [66] to strong simulation performance, is difficult to collect, partially due to farmer concerns about data privacy [68], limiting available information about commercial-sized plots. The availability of collocated crop growth model simulations allows us to (a) analyze the sensitivity of the genotype x management (G × M) factors retrieval by the satellite to variability in the relationship of satellite measurments and crop state variables and (b) use time-series analysis to analyze the uncertainty caused by this variability. Furthermore, the collocated crop growth model simulations are used to demonstrate the possibility of training and testing agricultural remote sensing algorithms with farmer-collected agromanagement data across a wide range of spatiotemporal variability, following the concept we introduced in [67] at the regional scale. Specifically, as in [67], the crop growth model simulations based on the provided data can be used to train and test remote sensing retrieval algorithms and, with sufficient farmer participation, a large swath of the spatiotemporal variability of the secondary factors affecting the retrievals can be covered. This dataset would allow further research to find methods to optimally use available weather, soil, and remote sensing data to create algorithms to map the regional-scale variability in G × E × M. As a result, by using crop growth model simulations at a fixed number of sites where the G × M parameters are known, a remote sensing retrieval algorithm could be trained to map G × M parameters where they are unknown and where no high quality collocated crop growth model simulations are available.

## 2. Materials and Methods

### 2.1. Data

In this study, we rely on two ground-truth maize datasets, which we term FLUX and LAIGROUND. The data sources are summarized in Table 2.

The FLUX dataset consists of CO_2_ flux tower measurements of gross primary productivity (GPP) and incoming solar radiation (SRAD) time series in maize fields. The eddy-covariance technique determines the CO_2_ flux, which is termed the net ecosystem exchange (NEE), from the covariance of the vertical wind velocity and CO_2_ flux, sampled by the tower at 10–20 Hz and averaged to 30–60 minute periods [69]. The height of the flux tower is selected to have an appropriate footprint covering the field being studied by the tower. The ecosystem respiration is removed from the NEE to obtain the amount of carbon captured by the producers in the field (GPP) by a partitioning algorithm. In this study, the GPP is either obtained from the nighttime-partitioned product provided by FLUXNET2015 [70] or the site principal investigators (PIs), or calculated from NEE using the nighttime-based partitioning algorithm of [71] implemented in [72]. In addition, ground-truth LAI that was measured at sites on some days of the season and the planting and harvest dates were obtained.

The LAIGROUND dataset consist of ground-truth LAI measurements of maize obtained during various campaigns with different measurement technique (Destructive, LAI2000, AccuPAR, Hemispheric Photography) compiled by [27]. Destructive measurements of LAI rely on physically sampling leaves in predefined areas in the field and measuring them in a laboratory to estimate the LAI in the field. In contrast, the LAI2000, AccuPAR, and Hemispheric Photography techniques use ground-based optical measurements made by researchers in the field on sampling campaign days, along with physics and image-processing based techniques, to estimate the LAI. Further details on all the different measurement techniques can be found in [73]. Each site in this dataset represents a different measurement campaign and some consist of LAI measurements on a single day in neighboring plots, some consist of LAI measurements in different fields (sometimes many kilometers apart), and some consist of multitemporal measurements in the same field/plot. Two of the sites are taken at CO_2_ eddy-covariance tower sites in the FLUX dataset (Italy and Mead) and the analysis conducted in this study takes care to ensure these are treated as the same sites across datasets when any site-based cross-validation-type analysis is conducted. Following [27], LAI measurments greater than 6 and less than 0.1 are excluded from the LAIGROUND dataset as they are beyond the prediction power of vegitation indicies.

In addition to the ground data in Table 2, we also use solar-reflective satellite data collocated with the ground data. Data from the Thematic Mapper (TM) sensor was used from LANDSAT 5, while data from the Enhanced Thematic Mapper Plus (ETM+) sensor was used from LANDSAT 7. The LANDSAT satellites used for each site depend upon which LANDSAT satellites were active when the site’s data was collected; LANDSAT 5 was active from March 1984–January 2013, while LANDSAT 7 was active from April 1999 to present (ca. August 2019). Data from both satellites was used at sites where data was collected when both satellites were active. For the LAIGROUND dataset, the plots tend to be small and we consequently use 30-m atmospherically-corrected LEDAPS surface reflectance data from LANDSAT 5 and 7 obtained from Google Earth Engine via the GEEXTRACT python tool within 5 m of the plot coordinates. For the FLUX dataset, the plots tend to be production-sized fields and we obtain the average LANDSAT LEDAPS [74] surface reflectance within a 100-m radius of the plot coordinates. In addition, because the LANDSAT temporal resolution is quite low, we obtain MODIS MCD43A4 BRDF-corrected nadir surface reflectance [75] at daily time steps (based on a weighted window of 16 days of measurements) at 500 m for the FLUX sites, allowing for temporal analysis of the retrieval performance. MODIS data was available for the entire study period for the FLUX sites.

### 2.2. Hybrid-Maize (HM) Simulations

Simulations from the Mead, Nebraska Ameriflux sites performed by [90] with the Hybrid-Maize (HM) crop growth model are used in this study. The simulations in [90] are based on accurate weather, soil, and agromanagement inputs at the sites and were publicly released [91]. The agromanagement inputs that were recorded at the sites and included in the simulations are planting date, cultivar maturity, plant density, and irrigation. The simulations were validated by [90] with respect to yield, crop respiration, soil respiration, and ecosystem respiration; they are further validated by us in Section 3.1 with respect to LAI and canopy light use efficiency (LUE_Canopy_).

### 2.3. Methods

In this subsection, we discuss the methods we use to evaluate the influence of site and growth stage specific secondary factors on the relationship between crop state variables and satellite measurments and the retrievability of G × M factors from satellite data. We focus on LAI and GPP in this study because these variables are some of the most commonly retrieved from remote sensing [92]. GPP also serves as a good complement to LAI because, unlike LAI, it is measured on a daily time scale at CO_2_ eddy-covariance tower stations. Thus, it can be used to provide validation of the temporal analysis performed on crop growth model simulations of LAI. In addition, it should be noted that, as in [67], the methods in this paper can be applied to crop growth model simulated variables whose time series are more difficult to measure than LAI and GPP, providing a basis to analyze performance over a wide range of crop state variables.

As daily GPP strongly depends on the daily SRAD, studies analyzing satellite-derived GPP must account for the strong temporal variability of SRAD when performing retrievals; this is because the variability in SRAD can mask the much smaller variability component in GPP caused by changes in the leaves, plants, and canopy structure [93]. A common technique to do so is correlating the product of the remote sensing measurement and SRAD with daily GPP, as opposed to the remote sensing measurement itself [93]. To achieve a result identical to [93], we analyze the canopy light use efficiency (LUE_Canopy_) in place of the GPP, which we define as

(1) $$LUE_{Canopy}\;=\;\frac{GPP}{SRAD},$$

As the definitions of various light use efficiencies are not standardized in the literature, we need to clarify that LUE_Canopy_ is essentially equivalent to LUE_Inc_ in [94], except that incident photosynthetically active radiation (PAR_inc_) is used in place of SRAD. In addition, we wish to note that for the purposes of this study, the criticism of LUE_Inc_ in [94] does not apply because our goal in calculating LUE_Canopy_ is simply to remove the influence of SRAD and not any plant-based process.

#### 2.3.1. Evaluation of HM Simulations

First, in order to use the HM simulations to evaluate the retrievals, we expand upon the validation performed by [90] to include LAI and LUE_Canopy_. To do so, the modeled and measured values are scatter plotted against each other and the coefficient of determination (R^2^) to the best-fit line and the root mean square error (RMSE) between the modeled and measured data are calculated. In order to facilitate comparison between the modeling performance of LAI versus LUE_Canopy_, only dates on which both LAI and LUE_Canopy_ measurements were available were included in the analysis to ensure that the distribution of crop growth stage did not vary between scatterplots or performance metrics (R^2^ and RMSE).

In addition, because daily LUE_Canopy_ measurements were available, a separate analysis of the performance of the LUE_Canopy_ values and the change in LUE_Canopy_ is made. The change in LUE_Canopy_ is defined as

(2) $$\Delta LUE_{Canopy}[t]\;=\;LUE_{Canopy}[t\;+\;\Delta \;-\;1]\;-\;LUE_{Canopy}[t\;-\;\Delta \;+\;1],$$

where Δ is in days and termed the Δ window. ΔLUE_Canopy_ is more sensitive to environmental-induced changes than the LUE_Canopy_ value itself and the performance in modeling it thus provides additional information on the strengths and limitations of the model.

Furthermore, because of high frequency variability in LUE_Canopy_, the time series modeling performance is analyzed at various levels of smoothing. The smoothing is performed by a moving average filter which is defined as

(3) $$\bar{LUE_{Canopy}}[t]\;=\;\frac{1}{2N\;-\;1}\sum_{i=-N+1}^{N-1}LUE_{Canopy}[t\;+\;i],$$

where *N* is in days and termed the smoothing window.

#### 2.3.2. Regression-Based LAI and LUE_Canopy_ Retrieval

Second, we train a regression of LANDSAT measurements to LAI and LUE_Canopy_ with the LAIGROUND and FLUX datasets. Specifically, we determine the regression coefficients in

(4) LAI = aEVI2 + b,

(5) $$LUE_{Canopy}\;=\;cEVI2\;+\;d,$$

where EVI2 is the Enhanced Vegetation Index 2 [27] and is defined as

(6) $$EVI2\;=\;2.5\frac{NIR\;-\;Red}{1\;+\;NIR\;+\;2.4Red},$$

and NIR is the surface reflectance in the near-infrared band, while Red is the surface reflectance in the red band. The NIR is designated as Band 4 (0.77–0.90 μm) on Landsat 5 and 7, while the Red is designated as Band 3 (0.63–0.69 μm). The coefficients are determined with leave-one-site-out cross-validation by calculating the coefficients on all sites except the one being evaluated. The RMSE performance is then assessed using the coefficients determined from all the other sites and the procedure is repeated for each site. In addition, confidence intervals for the coefficients are determined by bootstrapping. Specifically, for each left-out site, regression coefficients are determined for 1000 random subsets of the remaining sites with the probability of inclusion of a point in any individual random subset equaling 50%. The 5th and 95th percentiles for the regression coefficients of these subset realizations are used as the estimated lower and upper bound of the leave-one-out regression coefficients for the site.

The LAIGROUND and FLUX datasets are analyzed separately for this procedure. The nearest cloud-free LANDSAT measurement within 15 days of the ground measurement is used to analyze the LAIGROUND dataset for consistency with [27], while the average cloud-free LANDSAT measurement within 10 days of the ground measurement is used for the analysis of the FLUX dataset.

#### 2.3.3. Satellite Retrieval and Crop Growth Model Sensitivity Analysis

Third, we analyze the sensitivity of the crop growth model to its G × M inputs and analyze how uncertainty in the satellite retrieval of LAI propagates to the uncertainty in estimation of its G × M inputs. Specifically, we perform new Hybrid-Maize simulations based on the inputs used in [90], varying the planting density, the planting date, and the seed’s growing degree days to maturity from their actual values, and observe the error in the modeled LAI with respect to the measured LAI for the modified simulations. As the emergence date is directly input into the simulations in [90], a preliminary set of Hybrid-Maize simulations is used to determine the appropriate planting date in Hybrid-Maize for the observed emergence date and then this planting date is varied in the sensitivity analysis. This method of determining the planting date to be varied is used in place of the actual planting date to remove the uncertainty caused by modeling the planting to emergence time (as in [90]).

Comparison of the modeled LAI is performed with both the actual measured ground-truth LAI and the measured LAI retrieved from the MODIS measurements. To visualize the effect of the uncertainty in the regression coefficients, the error is shown for a range of regression coefficients determined from the confidence intervals obtained by bootstrapping in the previous subsection. Specifically, the slope of the regression is linearly varied from its minimum lower bound to its maximum upper bound while the intercept of the regression is simultaneously varied from its maximum upper bound to its minimum lower bound. As a large value for the intercept compensates for a lower value in the slope and vice versa, this method generates a realistic space within which to analyze the variation of the regression coefficients.

#### 2.3.4. Evaluation of Uncertainty of LAI and LUE_Canopy_ Retrievals Due to Site and Growth Stage Specific Factors with Temporal Analysis

Fourth, we assess the uncertainty of LAI and LUE_Canopy_ retrievals with temporal analysis due to site and growth stage specific factors. Due to the “one place, one time, one equation” concept [27], different regression equations should be used to retrieve the LAI and LUE_Canopy_ at different sites and growth stages (different times). Furthermore, data from different years may also appear to require different regression equations because the interannual difference in weather and agromanagement is very significant [13] and can cause large differences in secondary factors. Therefore, different years can also be considered different sites for the purposes of this analysis. In order to separate uncertainty caused by site and growth stage specific factors from other types of uncertainty, we use temporal analysis and focus on the retrieval of the temporal change in LAI and LUE_Canopy_. Errors caused by site and growth stage specific factors should be strongly positively correlated at the same place and nearby times; as a result, errors should partially cancel out when retrieving the temporal change as opposed to the actual values themselves. Thus, in order to assess the extent of the uncertainty caused by site and growth stage specific factors, the retrieval error of the change in LAI and LUE_Canopy_ is compared to the theoretical error of the change in LAI and LUE_Canopy_ assuming temporal independence of error.

To perform the temporal uncertainty analysis for LAI, we use the LAIGROUND dataset as the baseline retrieval and apply the LANDSAT-trained leave-one-site-out regression coefficients from Equation (4) to the MODIS MCD43A4 BDRF-adjusted daily surface reflectance time series to obtain retrievals of LAI with daily resolution. The NIR band is designated as Band 2 on MODIS (0.84–0.88 μm), while the Red band is designated as Band 1 on MODIS (0.62–0.67 μm). The training of the LAI retrieval algorithm is performed on the LAIGROUND dataset with LANDSAT measurements for two reasons:

- Using the LAIGROUND dataset with LANDSAT imagery better allows for the use of exact point measurements in fields and is thus less likely to be subject to uncertainty in training due to the inhomogeneity of LAI in the field, which can be significant [95].
- Training on high-resolution LANDSAT imagery as opposed to moderate-resolution MODIS imagery is preferable due to the significance of the mixed-pixel effect and neighboring pixels of other land types (including other crops) [95,96].

In addition, a scaling effect correction algorithm is not used to correct for the uncertainty in applying a regression trained on LANDSAT data to MODIS data as these algorithms generally require *a priori* information on the subpixel contents of the moderate resolution MODIS pixels [95,96] which is not readily available. For this reason, training on MODIS pixels would likely not provide a benefit with respect to the uncertainty as it is likely that the bias caused by LAI inhomogeneity and the mixed pixel effect varies strongly from site to site [95,96].

With these daily LAI retrievals from MODIS measurements, we calculated the change in LAI as

(7) ΔLAI[t] = LAI[t + Δ − 1] − LAI[t − Δ + 1],

where Δ is in days and termed the Δ window.

The MODIS-retrieved ΔLAI is compared to the crop growth model predicted ΔLAI using the correlation coefficient absolute value (|r|) and RMSE. These metrics are compared to the theoretical |r| and RMSE if the error of retrieved LAI [*t* + Δ − 1] and LAI [*t* − Δ + 1] were independent with a RMSE equivalent to the leave-one-site-out RMSE calculated in Section 2.3.2. In this case, the theoretical RMSE and |r| can be calculated as

(8) $$RMSE{\left(\Delta LAI[t]\right)}_{Theor}\;=\;RMSE(LAI[t\;+\;\Delta \;-\;1]\;-\;LAI[t\;-\;\Delta \;+\;1])\;=\;\sqrt{2}RMSE(LAI[t]),$$

(9) $$\left|r{\left(\Delta LAI[t]\right)}_{Theor}\right|\;=\;\left|\frac{cov\left(\Delta LAI_{actual}\;+\;e_{\Delta LAI},\;\Delta LAI_{actual}\right)}{\sqrt{var\left(\Delta LAI_{actual}\;+\;e_{\Delta LAI}\right)var\left(\Delta LAI_{actual}\right)}}\right|\;=\;\left|\frac{1}{\sqrt{1\;+\;{\left[\frac{\sqrt{2}RMSE(LAI[t])}{\sigma \left(\Delta LAI_{actual}\right)}\right]}^{2}}}\right|,$$

The uncertainty analysis for LUE_Canopy_ is complicated by the presence of high frequency components that need to be smoothed by Equation (3) in order to fully understand the temporal resolution of the retrieval. As the baseline retrieval methods with LANDSAT cannot account for the effects of the temporal smoothing because LANDSAT does not make daily measurements, the baseline retrieval must be retrained with MODIS measurements. Thus, leave-one-site-out regression is used to determine the regression coefficients in

(10) $$\bar{LUE_{Canopy}}\;=\;p\bar{EVI2}\;+\;q,$$

where $\bar{EVI2}$ is the moving average of EVI2 defined as

(11) $$\bar{EVI2}[t]\;=\;\frac{1}{2N\;-\;1}\sum_{i=-N+1}^{N-1}EVI2[t\;+\;i],$$

With these leave-one-site-out regression coefficients, a baseline RMSE for the retrieval of $\bar{LUE_{Canopy}}$ can be identified. In addition, as we have the benefit of a daily time series of MODIS measurements, $\bar{\Delta LUE_{Canopy}}$ (defined in the same way as ΔLUE_Canopy_ in Equation (2) can be determined by training a direct regression

(12) $$\bar{\Delta LUE_{Canopy}}\;=\;r(\bar{EVI2}[t\;+\;\Delta \;-\;1]\;-\;\bar{EVI2}[t\;-\;\Delta \;+\;1])\;+\;s,$$

in place of using Equation (10). The regression coefficients in Equation (12) are determined by leave-one-site-out cross-validation and the performance is compared to the theoretical |r| and RMSE performance defined in Equations (8) and (9) (with LUE_Canopy_ substituted for LAI). As using Equation (12) depends on having multiple sites for cross-validation, this analysis is only performed for the actual LUE_Canopy_ measurements, while only the |r| correlation with MODIS measurements is analyzed for the modeled measurements. The analysis for LUE_Canopy_ measurements is performed analyzed for the modeled measurements. The analysis for LUE_Canopy_ measurements is performed between the planting and harvest dates reported for the sites; the LUE_Canopy_ analysis is not performed at US-Bi2 due to the unavailability of planting and harvest dates at this site.

#### 2.3.5. Training LAI and LUE_Canopy_ Retrievals with HM Simulations

Lastly, in order to validate the concept of training and testing field-scale remote sensing retrievals with crop growth model simulations, we compare the performance of LAI and LUE_Canopy_ at sites other than those in Mead, Nebraska using (a) regression coefficients trained with the actual LAI and LUE_Canopy_ measurments at the Mead, Nebraska sites; and using (b) regression coefficients trained with HM modeled LAI and LUE_Canopy_ values at the Mead, Nebraska sites. These retrievals are trained and evaluated using LANDSAT measurements and the performance is reported site-by-site.

## 3. Results

### 3.1. Evaluation of HM Simulations

**Figure 1.**
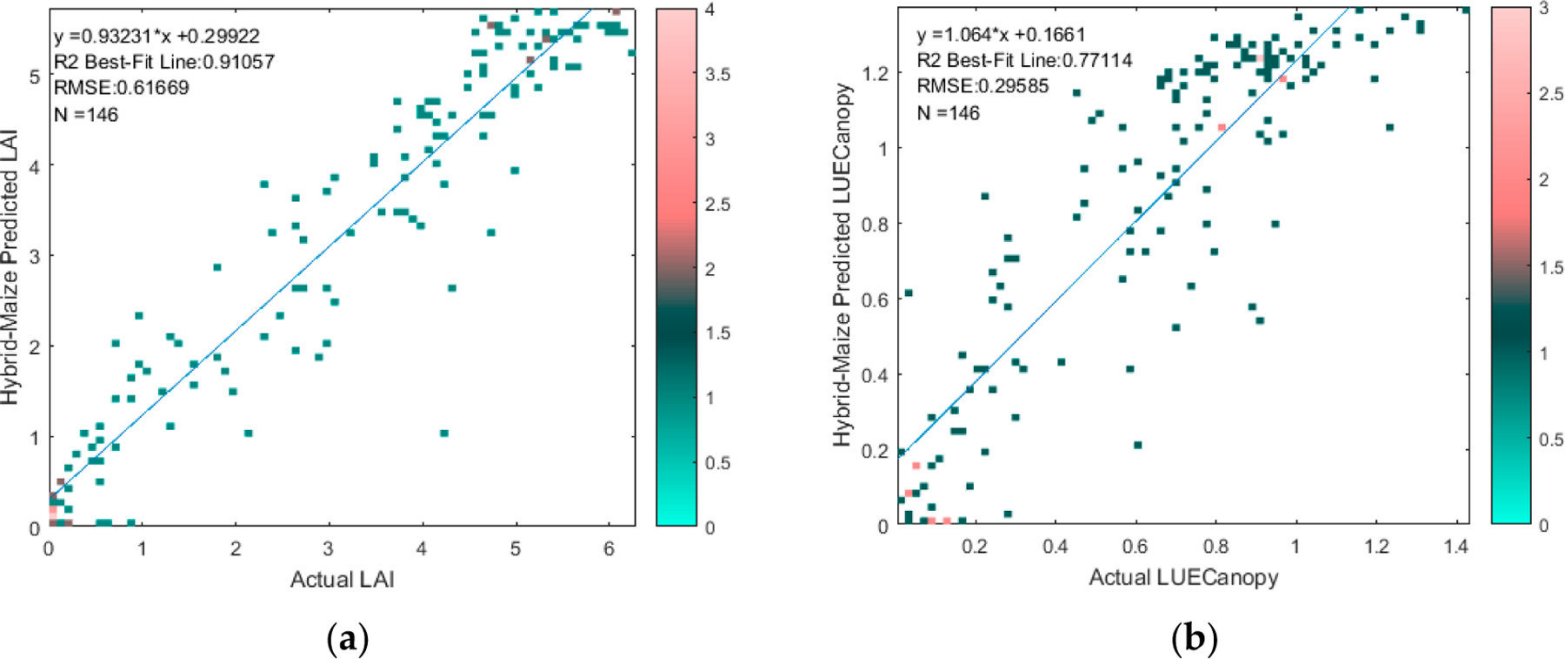
Comparison of actual versus Hybrid-Maize modeled (a) LAI and (b**)** LUE_Canopy._ The color bar represents the number of points at each marker on the scatter plot.

We first evaluate the performance at the Mead, Nebraska of the modeled HM LAI and LUE_Canopy_ at the Mead, Nebraska sites. In Figure 1a,b, we show scatterplots between the modeled HM LAI and LUE_Canopy_ values and the actual values on the ground. As discussed in Section 2.3.1, only dates that have both LAI and LUE_Canopy_ measurements are included in Figure 1a,b for consistent comparison of the modeling performance of these two variables. The figures show strong performance for modeled LAI and LUE_Canopy_ with R^2^ values of 0.91 and 0.77 and RMSE values of 0.62 and 0.30, respectively; although, the bias for LUE is relatively high.

**Figure 2.**
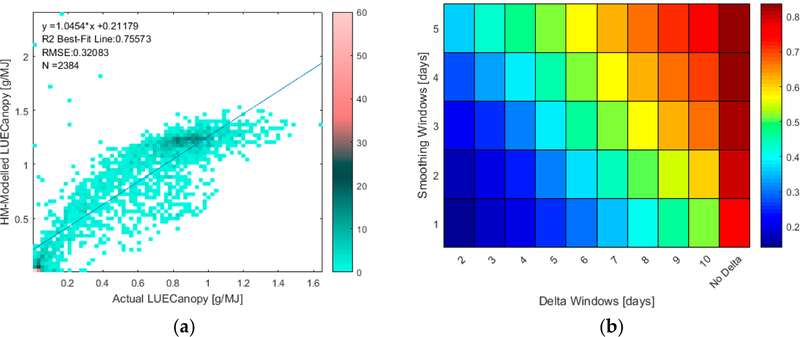
**(a)** Comparison of actual versus Hybrid-Maize modeled LUE_Canopy_. The color bar represents the number of points at each marker on the scatter plot. **(b)** R^2^ of actual versus Hybrid-Maize modeled LUE_Canopy_ and ΔLUE_Canopy_ at different levels of smoothing and values of Δ. N = 2384.

In Figure 2, the performance of modeled LUE_Canopy_ and ΔLUE_Canopy_ are shown for all ground measurements of LUE_Canopy_, not only those that also have a LAI measurement on the same date. Figure 2a shows the scatterplot of modeled LUE_Canopy_ versus actual LUE_Canopy_ with no smoothing, while Figure 2b shows the R^2^ value between modeled and actual LUE_Canopy_ and ΔLUE_Canopy_ at different levels of smoothing and values of Δ. As seen in Equation (3), a smoothing window of 1 represents no smoothing. Only days where modeled LUE_Canopy_ is greater than zero are included in Figure 2. In addition, a small number of days which have less than 95% of the underlying GPP time are series available not included in Figure 2.

The results in Figure 2 show that the performance of modeled LUE_Canopy_ is strong with an R^2^ of 0.76 in the absence of smoothing and slightly higher with smoothing. In contrast, as seen in Figure 2b, the performance of ΔLUE_Canopy_ is dependent on the level of smoothing and value of Δ, with stronger performance with longer Δ windows and more smoothing.

### 3.2. Regression-Based LAI and LUE_Canopy_ Retrieval

**Figure 3.**
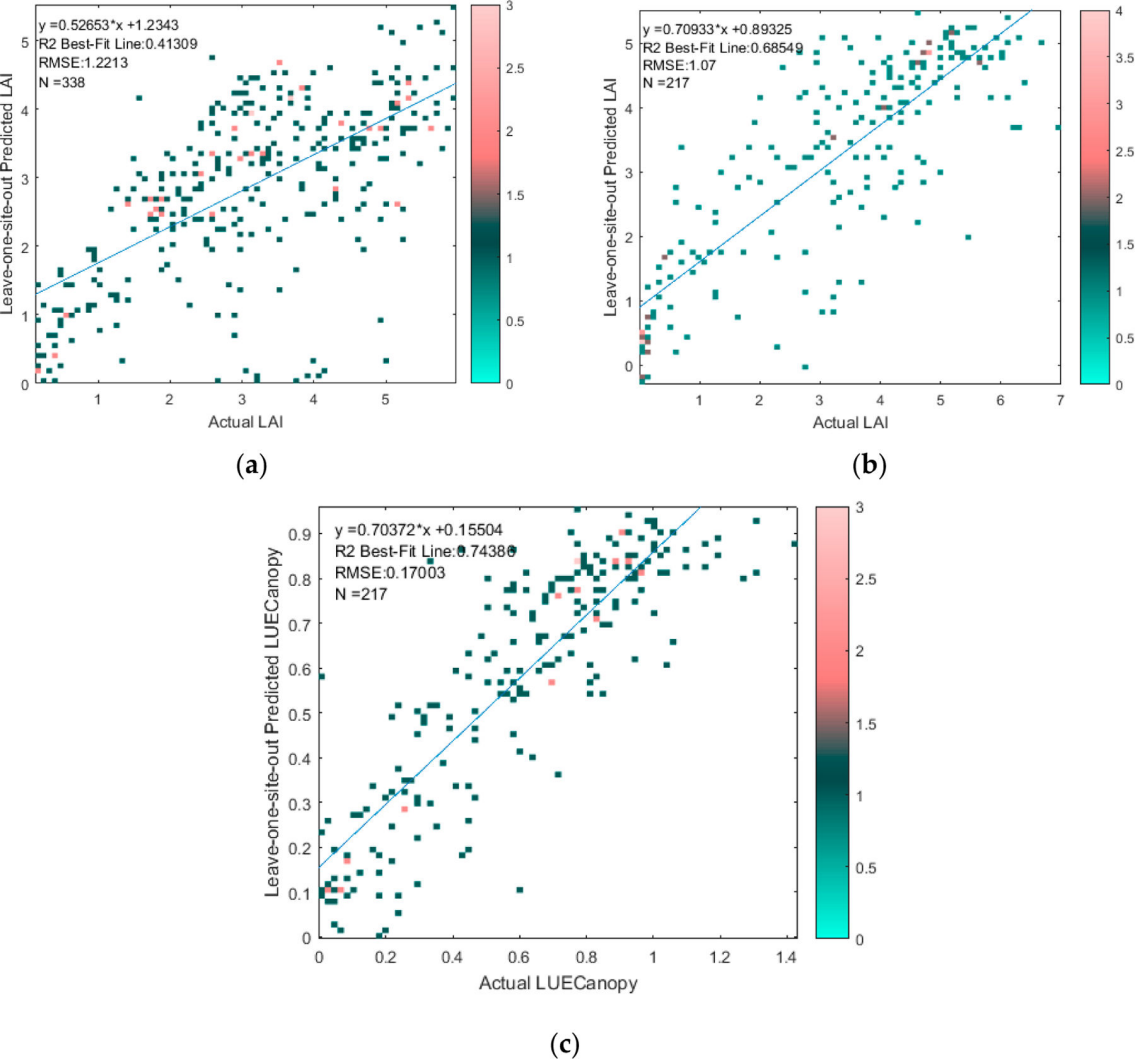
Comparison of retrieved versus actual (**a**) LAI from LAIGROUND dataset, (**b**) LAI from FLUX dataset, and (**c**) LUE_Canopy_ from FLUX dataset from LANDSAT measurements via leave-one-site-out cross validation. The color bars represent the number of points at each marker on the scatter plot.

We now present the results of the retrieval of LAI and LUE_Canopy_ from LANDSAT EVI2 by Equations (4) and (5) via leave-one-site-out cross validation. In Figure 3, we present the leave-one-leave-one-site-out performance for all sites combined in separate scatterplots for the LAIGROUND and FLUX datasets (prediction performed with leave-one-site-out site-by-site and then combined into a single scatter plot). Figure 3a shows the LAI retrieval scatterplot for the LAIGROUND dataset, while Figure 3b,c show the LAI and LUE_Canopy_ retrieval scatterplots for the FLUX dataset.

Figure 3 shows LAI retrieved with a R^2^ performance between 0.41 and 0.69 and an RMSE between 1.07 and 1.22, while LUE_Canopy_ is retrieved with an R^2^ performance of 0.74 and an RMSE of 0.17. In addition, the site-by-site leave-one-site-out retrieval performance and regression coefficients for the LAIGROUND dataset are shown in Table 3, while the corresponding information for the FLUX dataset is shown in Table 4. Tables 3 and 4 also show the confidence intervals for the determined leave-one-site-out coefficients.

### 3.3. Satellite Retrieval and Crop Growth Model Sensitivity Analysis

**Figure 4.**
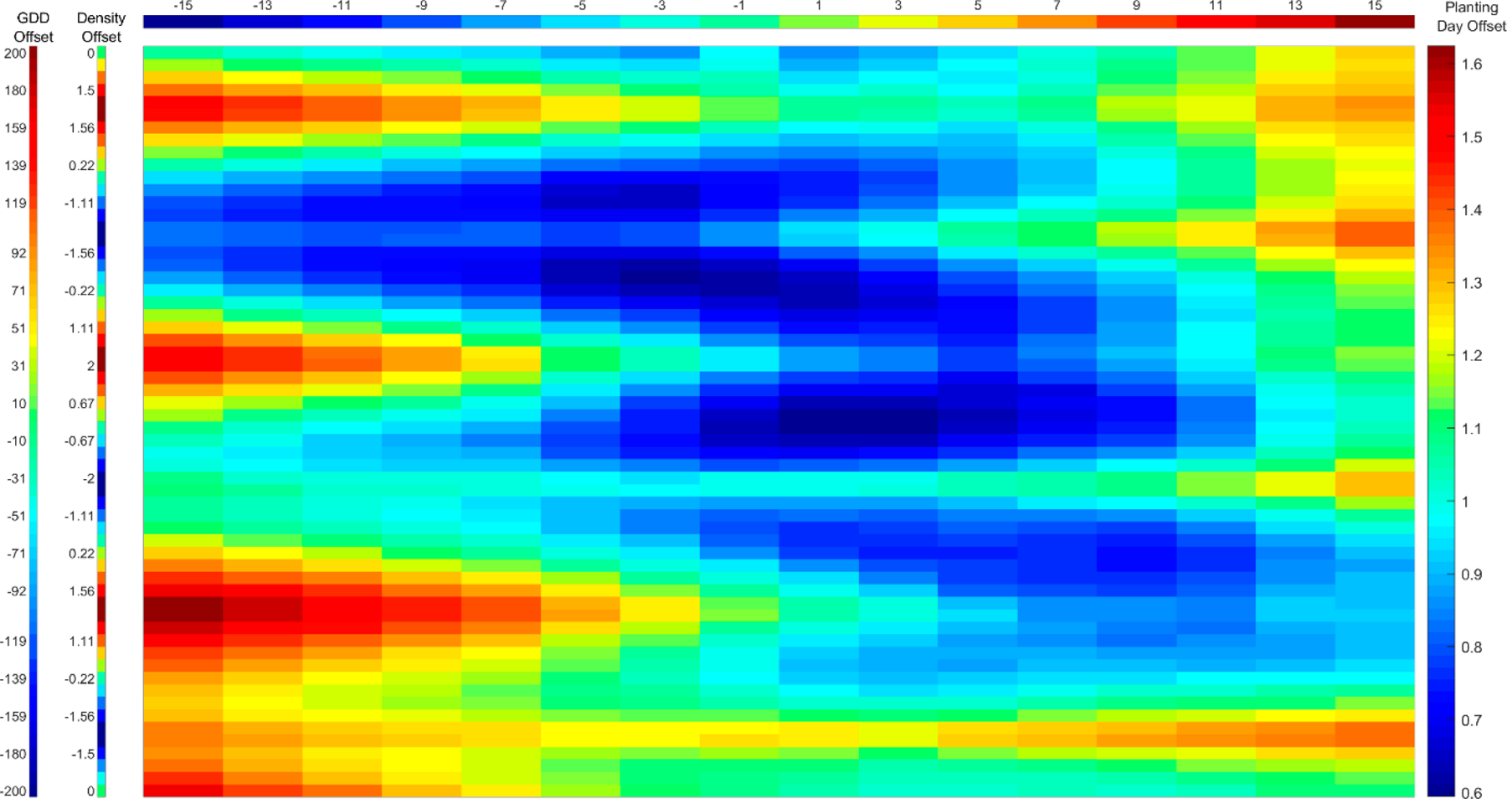
RMSE of modeled LAI with respect to ground-truth LAI while varying planting date, seed GDD to maturity, and planting density. Leftmost column represents offset from actual seed GDD to maturity in °C in simulation variant, while second leftmost column represents offset from actual planting density in plants/m^2^ in simulation variant. Header represents offset from actual planting day in days in simulation variant. Color bar at right and color in main panel represents LAI RMSE for each simulation variant determined by column and header. N = 146.

We now turn to presenting the results of the crop growth model-based sensitivity analysis. First, in Figure 4, we show the RMSE of the modeled LAI with respect to the actual ground truth LAI for different simulations where three G × M parameters (the planting date, seed GDD to maturity, and planting density) are o set by various amounts from their actual values. The results in Figure 4 allow for analysis of the effect of biases in combinations of the three G × M parameters varied in the figures. The results show that with respect to the ground-truth there are several combinations of parameter bias which lead to LAI RMSEs below 0.7 against the ground-truth measurements, demonstrating ill-posedness in the inversion of LAI values to G × M parameters. As expected, the situation where none of the parameters are biased (i.e., the actual G × M parameters applied in the field, at the center of the figure), leads to a low RMSE (near 0.6), however other combinations of biases have similar RMSE. The magnitude of the error seems to be most sensitive to variations in the planting density (as seen by patterns in the variation of the performance corresponding to the frequency of the density variation); however, significant negative GDD offsets and positive planting day delays are also seen to significantly increase the error. Overall, the error is highly variable with respect to the parameter biases and many combinations of biases lead to high error (a range of LAI RMSEs from 0.6 to 1.6 is observed). This variation shows the strong sensitivity of the LAI to these three G × M inputs and the interactions between them.

**Figure 5.**
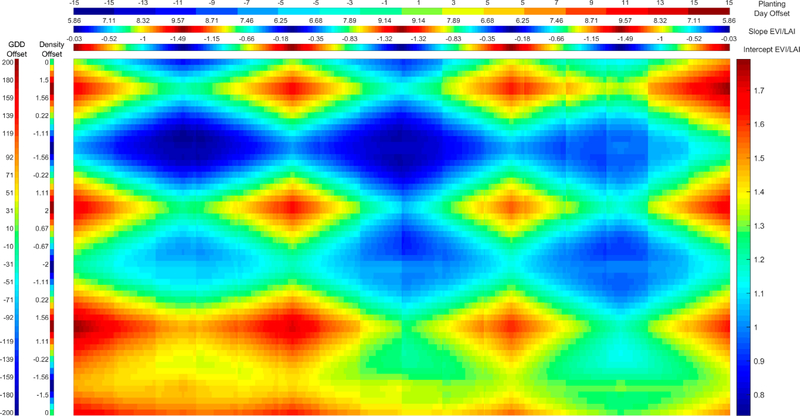
RMSE of modeled LAI with respect to MODIS-retrieved LAI while varying planting date, seed GDD to maturity, and planting density, and MODIS EVI2/LAI regression coefficients. Leftmost column represents offset from actual seed GDD to maturity in °C in simulation variant, while second leftmost column represents offset from actual planting density in plants/m2 in simulation variant. Topmost header represents offset from actual planting day in days in simulation variant. Second topmost header represents slope of EVI2/LAI regression coefficients. Third topmost header represents intercept of EVI2/LAI regression coefficients. Color bar at right and color in main panel represents LAI RMSE for each simulation variant determined by column and header. N = 3280.

In Figure 5, the sensitivity analysis from Figure 4 is reproduced with MODIS LAI retrievals instead of ground-truth LAI measurements. First, it is important to note that the analysis causes a great increase in the number of points analyzed (from N = 146 to N = 3280) and removes potential biases from a skewed distribution of growth stages as all dates are included, instead of just the dates where the ground-truth LAI measurements were taken. Secondly, the figure shows the change in modeled versus retrieved LAI error as the MODIS EVI2/LAI regression coefficients are varied. The results show the strong dependence of the error on both the regression coefficients used and the bias in the model parameters. Interestingly, although all regression coefficients show good performance for some combinations of G × M biases, some regression coefficients show significantly less sensitivity to G × M biases than others in terms of LAI error. For example, low regression slopes allow for low RMSE values at a limited number of G × M bias combinations, while high regression slopes allow for low RMSE values at a significantly greater number of G × M bias combinations. As in Figure 4, the variation in the LAI RMSE error is very sensitive to the variation of planting density, although negative GDD o sets also have a very significant effect in increasing the error. The ill-posedness of inverting the G × M factors from the MODIS measurements is seen clearly in the figure with several combinations of biases and regression coefficients leading to similar levels of LAI error. As expected, low parameter biases (near the center of the figure) lead to low LAI RMSE values, although negatively biasing the planting density appears to allow for better matchup with the MODIS measurements over a wider range of regression coefficients.

### 3.4. Evaluation of Uncertainty of LAI and LUE_Canopy_ Retrievals Due to Site and Growth Stage Specific Factors with Temporal Analysis

**Figure 6.**
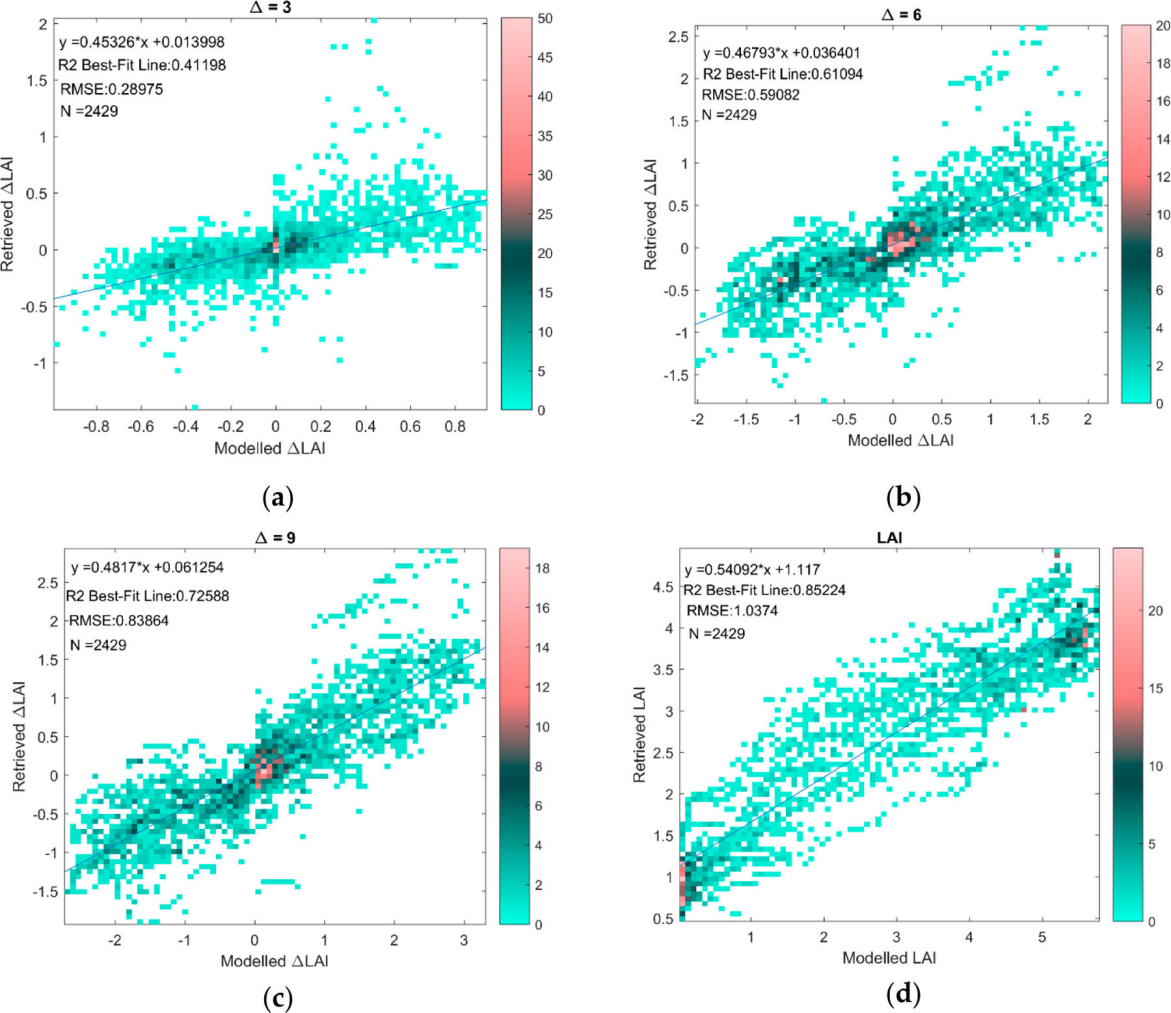
(**a**–**c**) Retrieved versus HM Modeled ΔLAI at Δ = 3, 6, 9; (**d**) Retrieved versus HM Modeled LAI. The color bars represents the number of points at each marker on the scatter plot.

We now present the results analyzing the uncertainty of LAI and LUE_Canopy_ retrievals due to site and growth stage specific factors with temporal analysis. First, in Figure 6, we show scatterplots of retrieved versus HM modeled ΔLAI at three values of Δ (Figure 6a–c, Δ = 3, 6, 9) and compare them to the retrieval performance of HM modeled LAI itself (Figure 6d). The leave-one-out regression values from Table 3 for Mead are used to perform the retrievals. The results in Figure 6 show a rising level of performance with increasing Δ values, ranging from an R^2^ of 0.41 for Δ = 3 to an R^2^ of 0.72 at Δ = 9. The retrieval of modeled LAI itself is seen to have an R^2^ of 0.85 in Figure 6d.

In Table 5, we show the actual and theoretical, modeled versus retrieved |r| and RMSE for LAI itself and ΔLAI for Δ = 2 to 10. The results in Table 5 show that the actual |r| and RMSE performance of the ΔLAI retrievals significantly outperform the theoretical performance calculated with Equations (8) and (9); for example, for Δ = 4, which corresponds to a week of measurements, the actual |r| and RMSE values are 0.70 and 0.40, while the theoretical values are 0.36 and 1.46, respectively.

**Figure 7.**
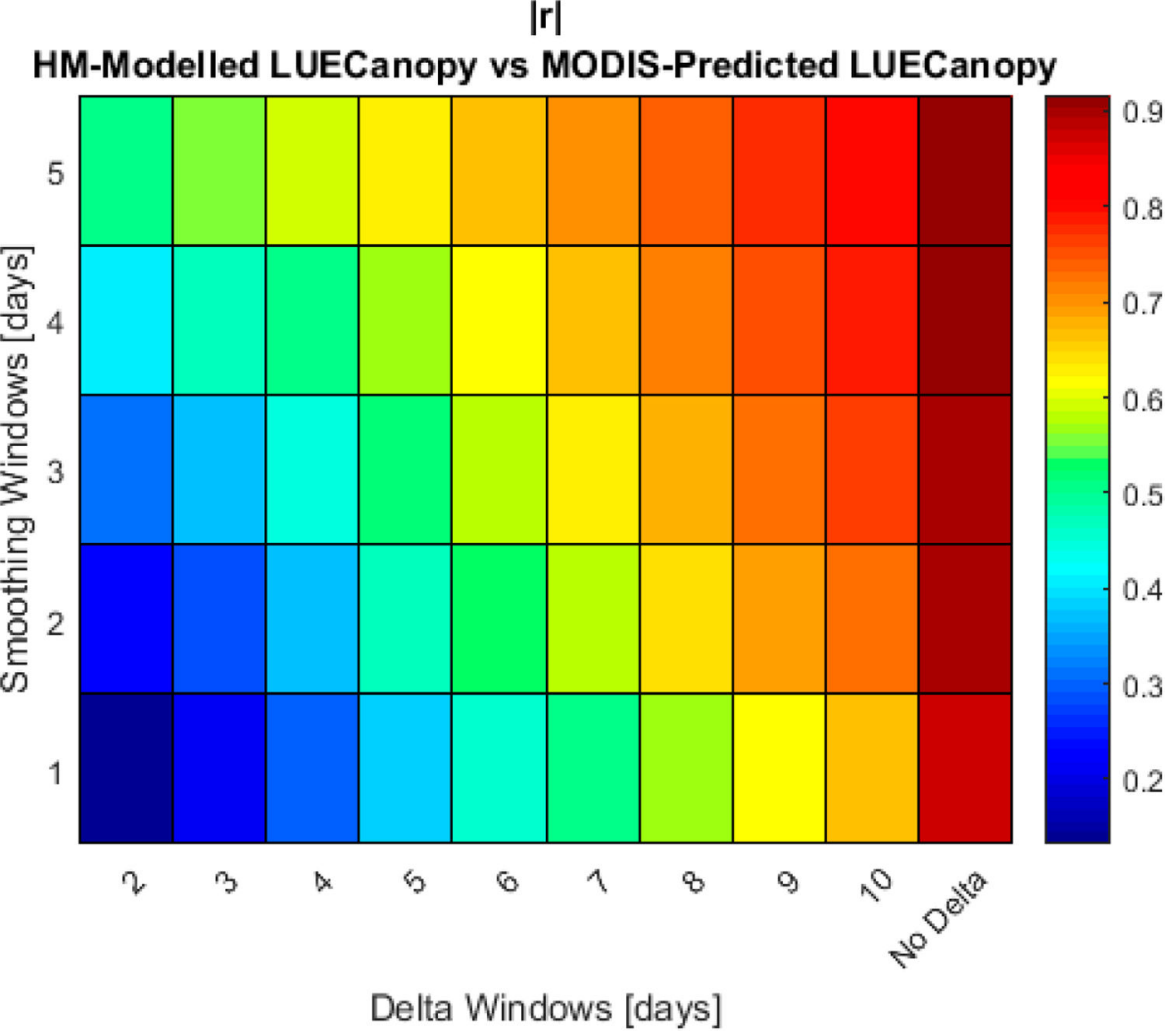
|r| correlation of the MODIS EVI2 measurements versus HM modeled ΔLUE_Canopy_ at different levels of smoothing and values of Δ. N = 2359.

In Figure 7, we present the |r| correlation of the MODIS EVI2 measurements versus modeled ΔLUE_Canopy_ at different levels of smoothing and values of Δ. The results in Figure 7 show that the |r| MODIS EVI2/ΔLUE_Canopy_ correlation strongly depends on the level of smoothing and the value of Δ; however, high |r| values may be obtained when smoothing is performed at Δ values above 5.

Only days with modeled LUE_Canopy_ greater than zero are included in Figures 6 and 7 and Table 5. In addition, for consistency with Figure 2, the small number of days which have less than 95% of the underlying measured GPP time series available are not included in Figure 7.

**Figure 8.**
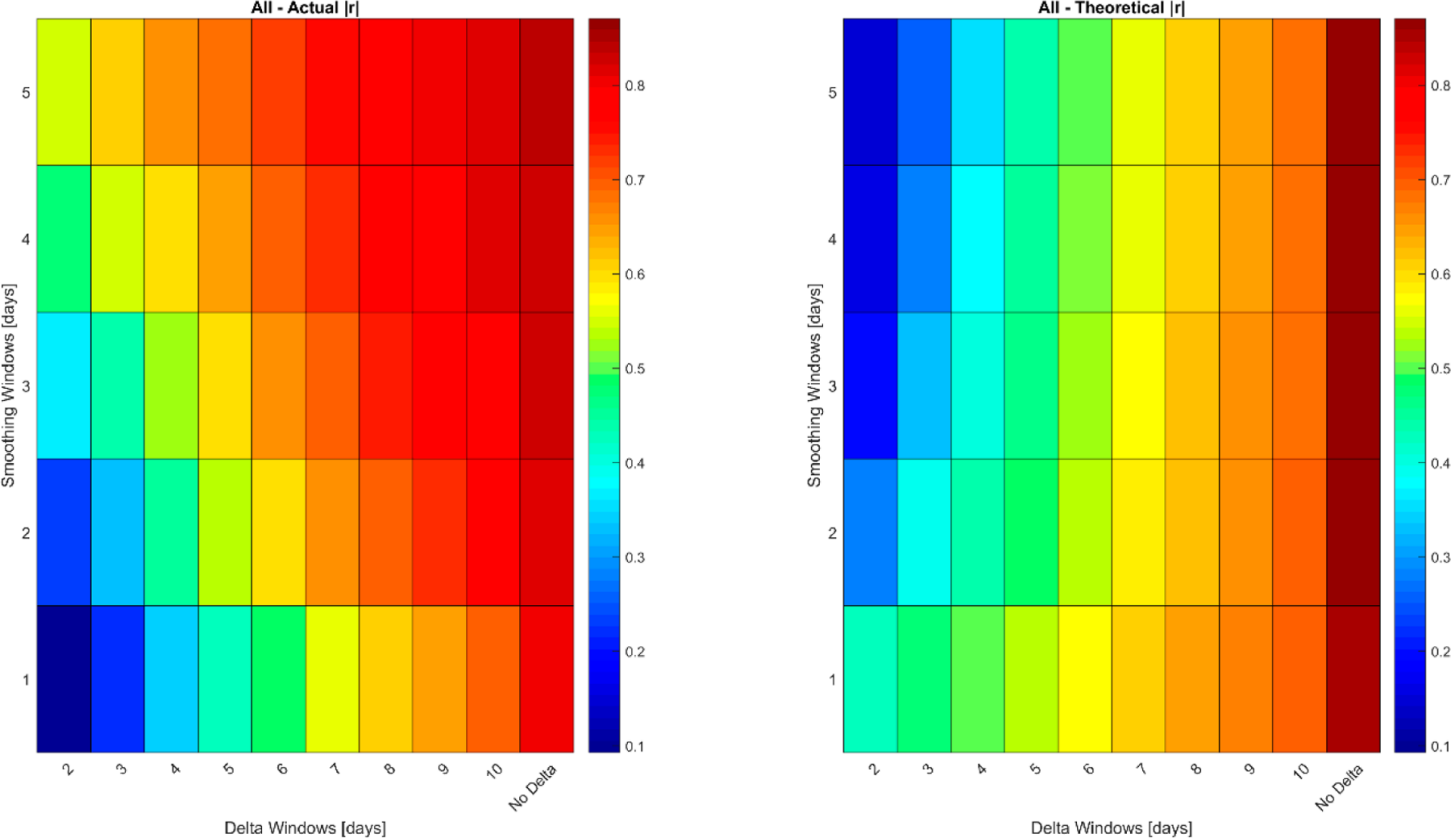
Actual versus theoretical |r| performance of the ΔLUE_Canopy_ retrievals at all sites at different levels of smoothing and values of Δ. N = 5071.

**Figure 9.**
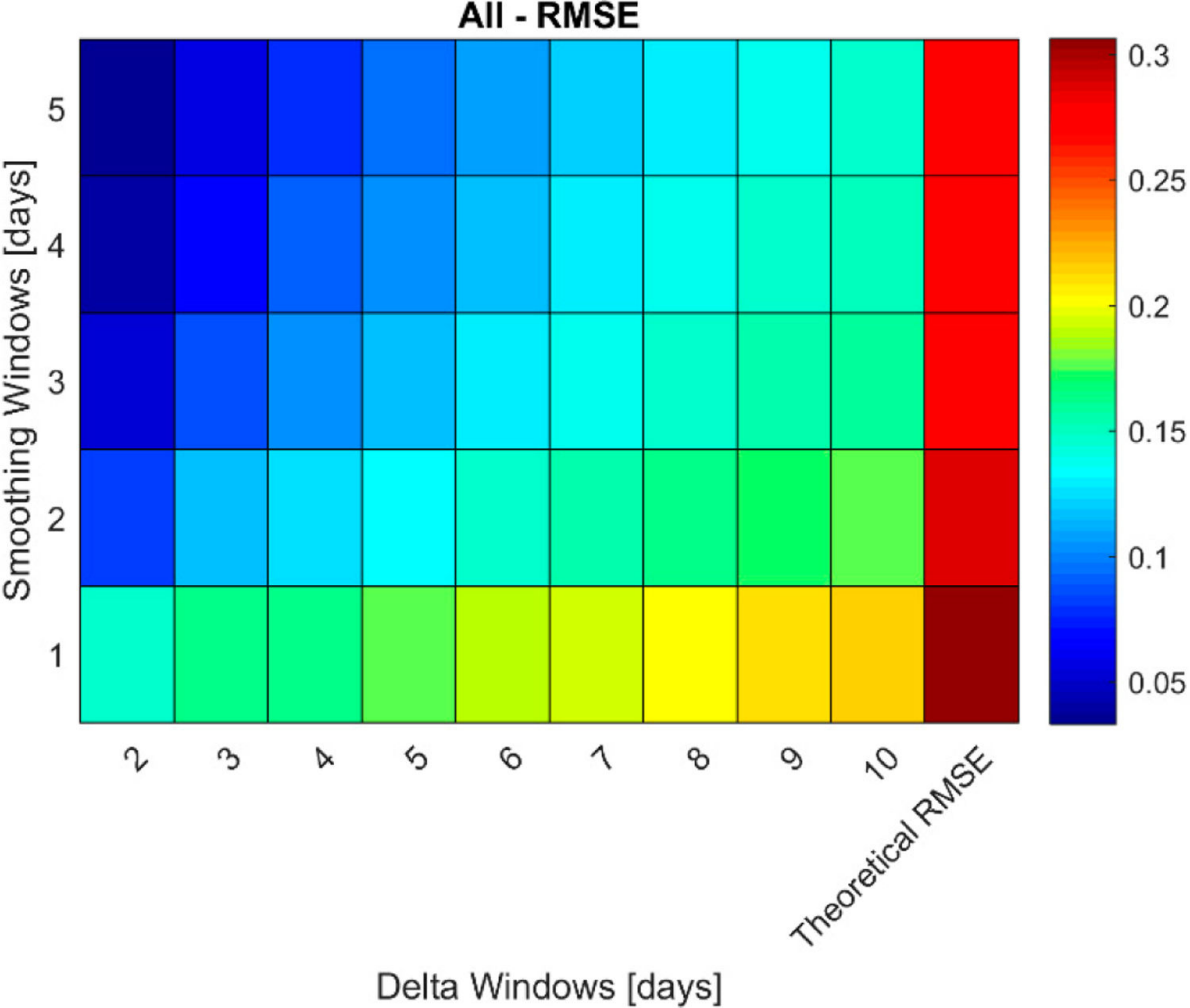
Actual versus theoretical RMSE performance of the ΔLUE_Canopy_ retrievals at all sites at different levels of smoothing and values of Δ. N = 5071.

In addition to comparison of modeled values (from the Mead, Nebraska sites) in Figure 7, ΔLUE_Canopy_ retrievals are compared against the actual ΔLUE_Canopy_ measurments at all the sites in the FLUX dataset. The performance against all the actual ΔLUE_Canopy_ data is shown in Figures 8 and 9, while the site-by-site performance is shown in Figures S1–S10 in the Supplementary Materials.

Figures 8 and 9 and Figures S1–S10 show that the actual ΔLUE_Canopy_ is retrieved with a performance at or above the theoretical performance assuming independence of retrieval error with respect to time and the relationship holds at most, but not all, sites. Poor performance at some sites, as seen in Figures S1–S10, may be explained by the large pixel size of MODIS (500 m), which can cause significant noise in the measurement of the daily time series depending on the size of the field and the inhomogeneity of the area surrounding the field [97,98]. Specifically, the mixed-pixel effect causes the signatures of neighboring pixels to be blended and it makes it difficult to separate the time-series of individual crops, especially if spring and winter crops are grown nearby [97], as is the case at some of the sites where poor performance is observed. Furthermore, the footprint of the flux tower measurements themselves depends on meteorological conditions and can be affected by process occurring the boundaries of the field [81,99]. Overall, however, strong performance is seen outside for the majority site-years of analyzed providing confidence in the retrievals.

### 3.5. Training LAI and LUE_Canopy_ Retrievals with HM Simulations

Lastly, we present the results indicating the performance of training the LAI and LUE_Canopy_ retrievals with HM modeled values as opposed to measured ground-truth values. In Table 6, we compare the RMSE of the LAI retrieval at sites other than Mead in the LAIGROUND dataset trained on either actual or modeled Mead LAI values, while in Table 7 we do the same for the LUE_Canopy_ retrievals in the FLUX dataset.

The results in Tables 6 and 7 show the difference in performance in using modeled versus actual data to train the LAI regression is small, while LUE_Canopy_ retrievals perform better when trained with actual, as opposed to modeled, values.

## 4. Discussion

The results presented in this study outline the importance of reducing the uncertainty in the relationship between satellite measurements and crop states variables caused by site and growth stage specific factors, in particular to use using remote sensing to map the G × E × M factors affecting crop growth. The importance of reducing the uncertainty is well illustrated by Figure 5, which shows that the set of allowable G × M parameters in terms of consistency with the MODIS LAI retrievals (as measured by the RMSE) is strongly a function of the regression coefficients chosen.

Based on the “one place, one time, one equation” concept [27], the appropriate regression coefficients for each time and place are ultimately different; therefore, auxiliary information is needed to select the appropriate regression coefficient column for each site and time to retrieve G × M in Figure 5. This variability of the regression coefficients is best seen on the LAIGROUND dataset with high-resolution LANDSAT measurements in both the coefficients themselves and the large confidence intervals in Table 3, from which the range of the regression coefficients in Figure 5 was constructed. Less variability is seen on the FLUX dataset in Table 4 because this dataset has fewer points, smaller diversity in sites (points from Mead, Nebraska make up more than half the dataset), and is not designed to test the spatial variability of LAI in nearby plots in the same way as the LAIGROUND dataset; as a result, the LAIGROUND results in Table 3 are more appropriate for analyzing the variability between sites. Analyzed in conjunction with Figure 5, the regression coefficient variability in Table 3 makes it very difficult to use remote sensing for mapping G × E × M. This is because, as illustrated in Figures 4 and 5, the retrieval of G × E × M is difficult due to equifinality (i.e., “multiple combinations of parameters leading to similar simulation accuracy”) [13] and, especially when the observations are uncertain remote sensing retrievals, is ill-posed. Figure 4 does a good job of showing the ill-posedness of the G × M retrieval even when using ground-truth LAI measurements; interestingly, due to the availability of the entire time series when using MODIS measurements in Figure 5, some combinations of G × M identified as probable in Figure 4 are not probable in Figure 5 for any combination of regression coefficients. This is an illustration of the importance of the number of measurements [13,100] needed to perform G × E × M retrievals and the frequent, low-cost observations provided by satellites may be one of the most promising technologies to achieve that goal [101].

Although the uncertainty caused by site and growth stage specific secondary factors is well-known [27–29,50,51], it is difficult to isolate it from other sources. One approach to understand it [29,50,51] is to include variables connected to the secondary factors that cause it in the regression methodology. Unfortunately, this approach requires that the secondary factors causing the uncertainty are known and recorded or measured prior to the analysis being conducted. As a result, these studies can miss some of the factors causing the issue and underestimate its extent. Another approach is to train a global relationship between the satellite variables and crop state variables, ignoring the secondary factors [27]. In this case [27], the issue is seen from the variability of the regression coefficients, as in our analysis in Tables 3 and 4, as well as indirectly from the variability in the leave-one-site-out RMSE error. However, this method cannot be used to exclude other sources of uncertainty from the retrieval, such as random error and the mixed-pixel effect [102].

In this study, temporal analysis is used to avoid these alternate sources [27,29,50,51] of uncertainty in determining the portion caused by site and growth stage specific secondary factors. The results in Table 5 show that the modeled ΔLAI is retrieved from the MODIS measurements with significantly better performance in terms of both |r| and RMSE as compared to the theoretical values assuming temporal independence of error, indicating significant site and time correlation of error. These results are also reproduced with actual ΔLUE_Canopy_ measurements across multiple CO_2_ flux tower sites in Figures 8 and 9. Both the results with modeled ΔLAI and actual ΔLUE_Canopy_ indicate a significant portion of the error can be removed by either predicting the secondary factors [29,50,51] or developing better methods to remove their influence, such as identifying vegetation indices less sensitive to the secondary factors [32,45,46]. The difference between the actual and theoretical |r| and RMSE for both ΔLAI and ΔLUE_Canopy_ provide an indication of the possible reduction in uncertainty by addressing the issue with secondary factors. The change in performance with respect to the value of Δ is driven by two factors:

- As Δ increases, the correlation between the error in the retrieved LAI or LUE_Canopy_ at t_2_ relative to t_1_ decreases because the measurements are more likely to be in different growth stages.
- As Δ increases, the magnitude of the retrieved ΔLAI or ΔLUE_Canopy_ increases relative to the remaining error which is not cancelled when calculating the change in the retrieved variables from the variables themselves, i.e., e[t_2_] – e[t_1_].

As a result of these opposing error-influencing forces, a single value for the improvement in the performance that could be obtained by reducing the influence of the secondary factors cannot be reported; however, as seen from Table 5 and Figures 8 and 9 the improvement can be quite dramatic. For example, for ΔLAI, the actual |r| at Δ = 2 is 0.52 (compared to a theoretical value of 0.13), while the actual |r| at Δ = 5 is 0.75 (compared to a theoretical value of 0.45).

Furthermore, the retrieval of ΔLAI and ΔLUE_Canopy_ is also useful as a measure of the timescale of the sensitivity of the MODIS measurements to changes in the canopy structure and crop status. Good responsiveness to time-sensitive processes is important for several applications of crop remote sensing. For example, good responsiveness is important in monitoring phenology/crop growth stage [103–105], in-season detection of nitrogen [106,107], water [107], and disease [108] stresses, and measurement of change in canopy structure during important growth stages [109]; these applications have proven useful in crop growth modeling [59], precision agriculture [110], and phenotyping for breeding selection [109], respectively. Our results show that satellite measurements can be used to detect changes in LAI and LUE_Canopy_ faster and with higher accuracy than would be expected if the error in LAI and LUE_Canopy_ retrievals were not autocorrelated in time. As a result, we also show the potential to rapidly detect growth and stress related changes in crop state variables with greater precision than that would be inferred from looking at generic performance validation studies [27,28].

The analysis used in this study relied on strong crop growth model simulation performance to expand the dataset of ground-truth LAI values to daily resolution. The strong performance of the HM simulations at the Mead, Nebraska sites, seen in [90] and Figures 1 and 2, provides a potential path [67] for future research to expand the development of testing agronomic satellite retrievals to a wide variety of G x E x M factors with farmer-provided agromanagement data. The results in Table 6 show that using HM simulation data from Mead, Nebraska to train LAI retrievals can provide nearly identical performance to using actual ground-truth LAI measurements from Mead, while Table 7 shows there are some relatively significant biases in using modeled LUECanopy to perform the training. The results for training LAI retrievals on HM simulation data show the potential of using farmer-provided agromanagement data to train, test, and improve retrieval algorithms, although a significantly greater number of sites is needed to understand the generalizability and biases in this approach. Nevertheless, the potential of reducing the uncertainty in the retrieval of crop state variables and the potential to map G × E × M factors shown in this study provides strong support for pursuing this collocated crop growth model simulation approach in agricultural remote sensing and should encourage researchers to increase their collaborative efforts with farmers [68,111].

## 5. Conclusions

Overall, this study showed that the uncertainty in the relationship between satellite measurements and crop state variables caused by site and growth stage specific factors is significant and that this uncertainty leads to significant difficulties in using remotely sensed data to retrieve the genotype × environment × management (G × E × M) factors affecting crop growth. Specifically, we performed an extensive temporal analysis and retrieved the temporal change in the state variables to show the amount of uncertainty caused by this secondary factor variability. We also conducted a joint sensitivity analysis of the remote sensing regression parameters and crop model genotype x management (G × M) parameters to illustrate the ill-posedness of retrieving G × E × M factors from satellite measurements. This analysis demonstrated the criticalness of reducing the uncertainty in the relationship between satellite measurements and crop state variables to make the retrieval more feasible. The study shows the need for further data collection and model development that can ultimately lead to methods that will minimize the secondary uncertainty caused by site and growth stage specific factors. In addition, further work needs to be conducted to address the application of the methods to use training data in regions where biotic stresses are poorly controlled and where, unlike the case in highly developed commercial agriculture systems [7], crop growth models show significant uncertainties in predicting actual yields (as opposed to potential yields) due to suboptimal management [112]. This research is critical to achieving the goal of mapping G × E × M factors on a global scale, which can improve our ability to make predictions about the global agricultural system [113].

## Supplementary Material

S001

## Figures and Tables

**Table 1.** Examples of common G × E × M factors included in crop growth model simulations [2,3].

Genotype (G)	Environment (E)	Management (M)
	-Air temperature	
-Relative maturity/Growing degree days (GDD) to maturity	-Precipitation	-Planting date
	-Solar radiation	-Planting density
-GDD to flowering	-Soil bulk density	-Fertilization
-Potential kernel number per ear	-Soil available water	-Irrigation
-Grain growth rate	-Soil organic matter	
	-Soil pH	

**Table 2.** Ground-truth data sources.

Name	Source(s)	Sites			Variables	
		Name	Latitude	Longitude	Name	Years
Flux Tower Data (Dataset FLUX)	Ameriflux [76]	US-Ne1 [35]	41.17	−96.48	GPP SRAD Ground-truth LAI Planting Date Harvest Date	2001–2009
		US-Ne2 [35]	41.16	−96.47		2001–2009, odd years
		US-Ne3 [35]	41.18	−96.44		2001–2009, odd years
		US-Ro1 [77]	44.71	−93.09		2005, 2009, 2011, 2013
		US-Bi2 [78]	38.11	−121.54		2017–2018
		US-ARM [79]	36.61	−97.49		2008
	GHG Europe	DE-Kli [80]	50.89	13.52		2007, 2012
		FR-Gri [81]	48.84	1.95		2008, 2011
		FR-Lam [82]	43.5	1.24		2006, 2008, 2010
		IT-BCi [83]	40.52	14.96		2004–2009
		NL-Lan [84]	51.95	4.90		2005
LAI Validation Data (Dataset LAIGROUND)		Beltsville	39.02	−76.85	Ground-truth LAI	1998 (N = 26)
		CEFLES2 [85]	44.37–44.46	0.19–0.41		2007 (N = 26)
		California [86]	35.48–39.22	−122.14–119.28		2011–2012 (N = 59)
		Italy (IT-BCi) [83]	40.52	14.96		2008–2009 (N = 35)
	[27]	Mead (US-Ne1 to US-Ne3) [35]	41.16	−96.46		2001–2012 (N = 92)
		Missouri [87]	39.22	−92.12		2002 (N = 10)
		NAFE06 [88]	−35.08–34.65	145.87–146.3		2006 (N = 14)
		SEN3EXP2009 [85]	39.02–39.08	−2.13–2.08		2009 (N = 10)
		SMEX02-IA [89]	41.76–42.67	−93.73–93.28		2002 (N = 21)
		SPARC [85]	39.03–39.15	−2.18–1.88		2003–2004 (N = 45)

**Table 3.** Leave-one-site-out LAIGROUND LANDSAT regression retrieval performance using Equation (4). a and b are the leave-one-site-out regression coefficients defined in Equation (4).

			Best-Fit		Lower Bound		Upper Bound	
			Coefficients		Confidence Interval		Confidence Interval	
Site Name	LAI RMSE	N	a	b	a	b	a	b
Beltsville	0.85	26	8.41	−0.92	7.73	−1.18	8.94	−0.65
CEFLES2	0.60	26	8.55	−1.04	7.76	−1.31	9.10	−0.79
California	1.32	59	8.19	−1	7.60	−1.43	9.22	−0.77
Italy	1.58	35	8.49	−1.20	7.82	−1.49	9.33	−0.92
Mead	1.03	92	7.27	−0.71	5.86	−0.9	7.67	−0.03
Missouri	0.98	10	8.13	−0.87	7.57	−1.18	8.81	−0.64
NAFE06	0.31	14	8.08	−0.85	7.50	−1.42	9.19	−0.61
SEN3EXP2009	0.89	10	8.20	−0.94	7.61	−1.26	8.90	−0.77
SMEX02-IA	1.23	21	8.66	−1.06	8.03	−1.35	9.27	−0.83
SPARC	1.74	45	9.17	−1.31	8.67	−1.55	9.73	−1.03

**Table 4.** Leave-one-site-out FLUX LANDSAT regression retrieval performance using Equations (4) and (5). a, b, c, and d are the leave-one-site-out regression coefficients defined in Equations (4) and (5).

	RMSE			Best-Fit Coefficients				Lower Bound Confidence Interval				Upper Bound Confidence Interval			
Site	LAI	LUE	N	a	b	c	d	a	b	c	d	a	b	c	d
DE-Kli	0.85	0.20	4	9.52	−1.24	1.67	−0.16	9.29	−1.36	1.57	−0.20	9.85	−1.11	1.75	−0.13
FR-Gri	2.83	0.18	1	9.52	−1.24	1.67	−0.16	9.28	−1.36	1.58	−0.20	9.88	−1.09	1.76	−0.14
FR-Lam	1.11	0.20	16	9.64	−1.25	1.68	−0.17	9.40	−1.38	1.61	−0.21	9.96	−1.15	1.77	−0.15
IT-Bci	1.41	0.18	32	9.50	−1.27	1.69	−0.17	9.28	−1.39	1.62	−0.22	9.83	−1.15	1.80	−0.15
US-Arm	0.14	0.23	1	9.52	−1.24	1.66	−0.16	9.24	−1.36	1.57	−0.19	9.87	−1.03	1.74	−0.13
US-Bi	1.63	0.26	12	9.52	−1.25	1.66	−0.16	9.35	−1.40	1.57	−0.20	9.90	−1.17	1.74	−0.13
US-Ne	0.83	0.16	124	8.84	−0.80	1.44	−0.09	5.08	−0.96	1.11	−0.18	9.62	1.36	1.68	0.07
US-Ro	1.16	0.13	27	9.59	−1.20	1.65	−0.16	9.25	−1.37	1.51	−0.18	9.93	−1.03	1.71	−0.10

**Table 5.** Comparison of HM modeled versus retrieved, actual and theoretical |r| and RMSE for the retrieval of ΔLAI and LAI at different values of Δ.

Δ (Days)	|r|-Modeled v Retrieved	|r|-Modeled v Retrieved Theoretical	RMSE-Modeled v Retrieved	RMSE-Modeled v Retrieved Theoretical	N
2	0.52	0.13	0.17	1.46	2429
3	0.64	0.25	0.29	1.46	2429
4	0.70	0.36	0.40	1.46	2429
5	0.75	0.45	0.50	1.46	2429
6	0.78	0.53	0.59	1.46	2429
7	0.81	0.59	0.68	1.46	2429
8	0.83	0.65	0.76	1.46	2429
9	0.85	0.69	0.84	1.46	2429
10	0.87	0.73	0.91	1.46	2429
Value Itself (no delta)	0.92	0.88	1.04	1.03	2429

**Table 6.** Comparison of LAI retrieval performance on all sites except Mead, Nebraska in LAIGROUND dataset trained with actual and HM-modeled Mead, Nebraska LAI values. Only sites with ≥ 10 points listed site-by-site; all points included in last row.

Site	N	RMSE Trained with Actual Data	RMSE Trained with Modeled Data
Beltsville	26	0.84	0.97
CEFLES2	26	0.77	0.87
California	59	1.40	1.39
Italy	35	1.39	1.26
Missouri	10	0.62	0.78
NAFE06	14	0.51	0.47
SEN3EXP2009	10	0.87	0.79
SMEX02-IA	21	1.20	1.32
SPARC	45	1.87	1.83
**All except Mead, Nebraska**	267	1.30	1.29

**Table 7.** Comparison of LUE_Canopy_ retrieval performance on all sites except Mead, Nebraska in FLUX dataset trained with actual and HM-modeled Mead, Nebraska LUE_Canopy_ values.

Site	N	RMSE Trained with Actual Data	RMSE Trained with Modeled Data
DE-Kli	4	0.20	0.20
FR-Gri	1	0.20	0.10
FR-Lam	16	0.21	0.29
IT-BCi	32	0.19	0.35
US-ARM	1	0.22	0.37
US-Bi2	12	0.26	0.30
US-Ro1	27	0.13	0.28
**All except Mead, Nebraska**	93	0.19	0.31
