# Supplementary material for: Evaluation of the Uncertainty in Satellite-Based Crop State Variable Retrievals Due to Site and Growth Stage Specific Factors and Their Potential in Coupling with Crop Growth Models
Source: Remote Sens (Basel). Author manuscript; Available in PMC 2019 Sep 18. (PMC6750221; doi:10.3390/rs11161928)
Supplement: S001 [file NIHMS1047113-supplement-S001.pdf]

Supplementary Materials: Evaluation of the Uncertainty in Satellite-based Crop State Variable Retrievals Due to Site and Growth Stage Specific Factors and Their Potential in Coupling with Crop Growth Models *Remote Sensing* 2019, *8*, remote sensing-567576.

Nathaniel Levitan [1,*], Yanghui Kang [2,3], Mutlu Özdoğan [3,4], Vincenzo Magliulo [5], Paulo Castillo [6], Fred Moshary[1] and Barry Gross [1]

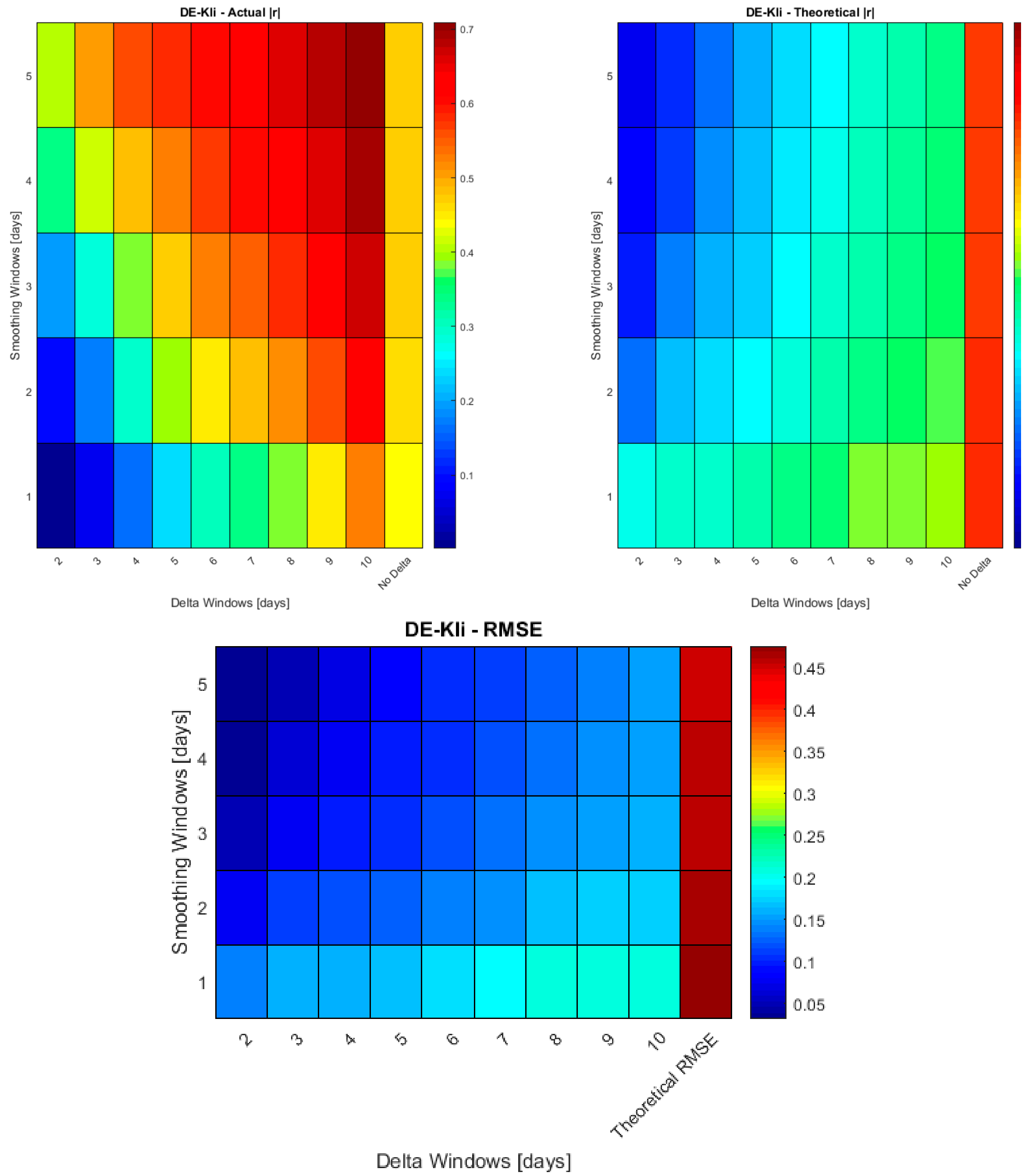

Figure S1. Actual versus theoretical |r| and RMSE performance of the ΔLUE_{Canopy} retrievals at DE-Kli at different levels of smoothing and values of Δ. N = 256.

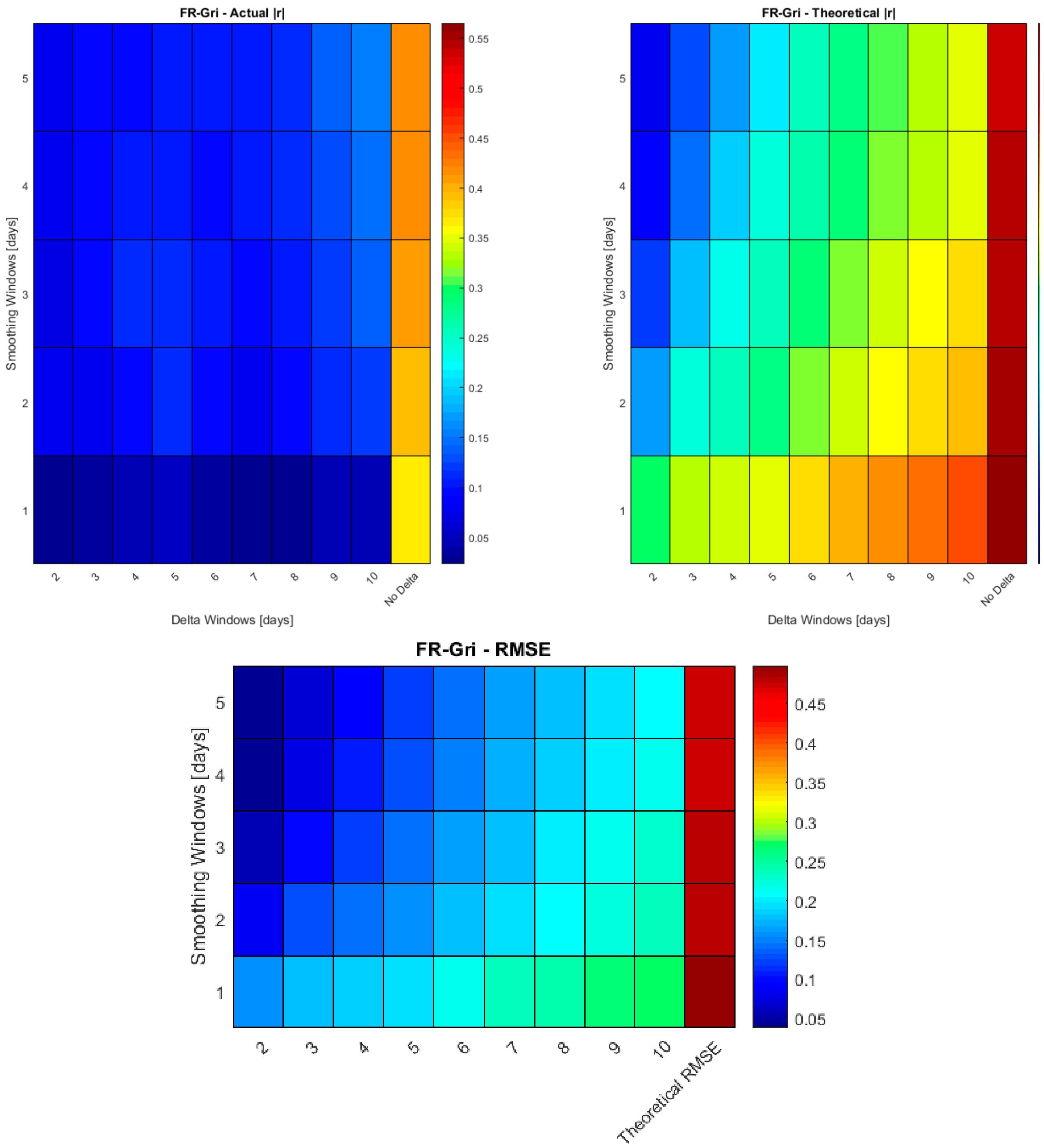

Figure S2. Actual versus theoretical |r| and RMSE performance of the ΔLUE_{Canopy} retrievals at FR-Gri at different levels of smoothing and values of Δ. N = 258.

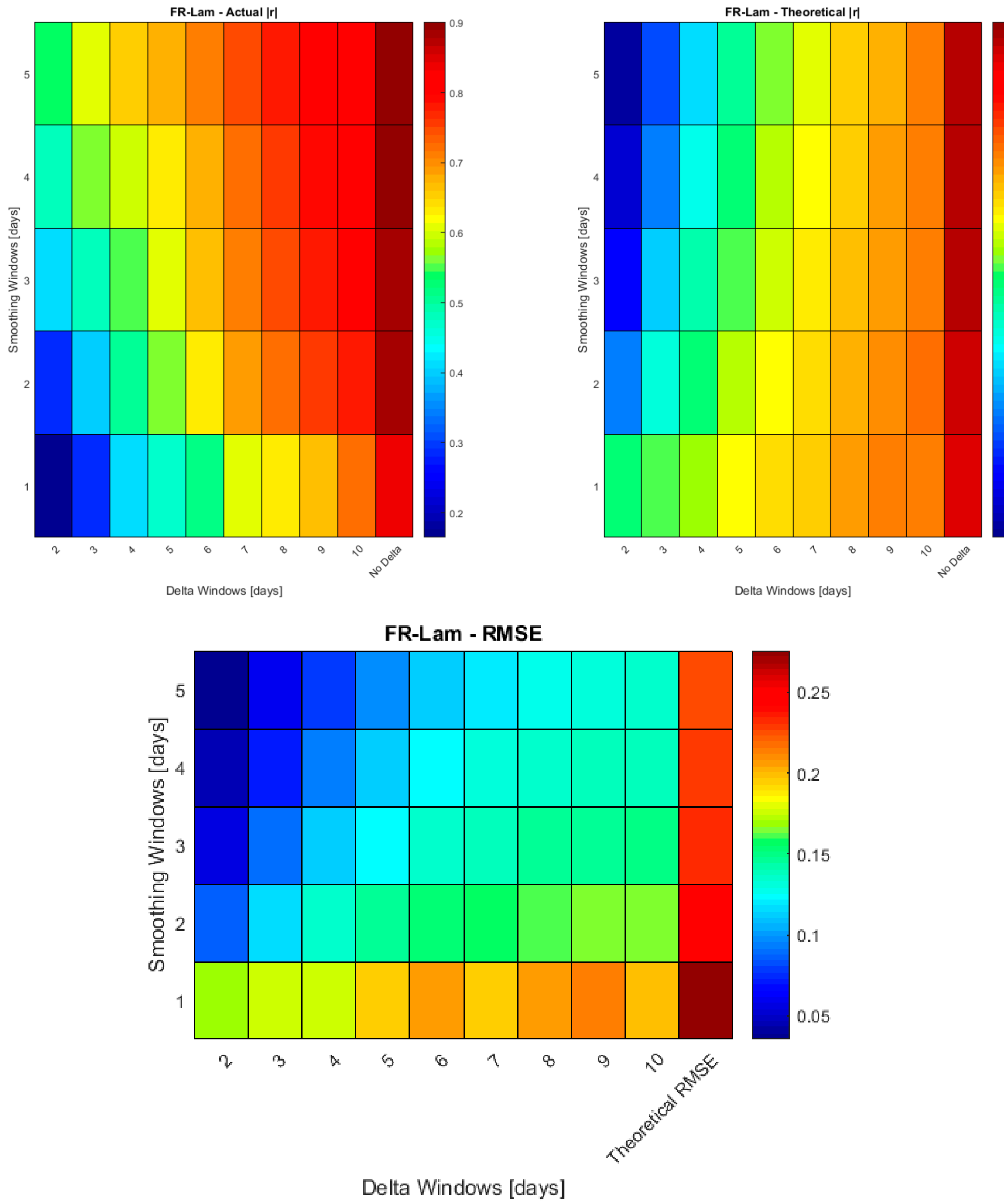

Figure S3. Actual versus theoretical |r| and RMSE performance of the ΔLUE_{Canopy} retrievals at FR-Lam at different levels of smoothing and values of Δ. N = 461.

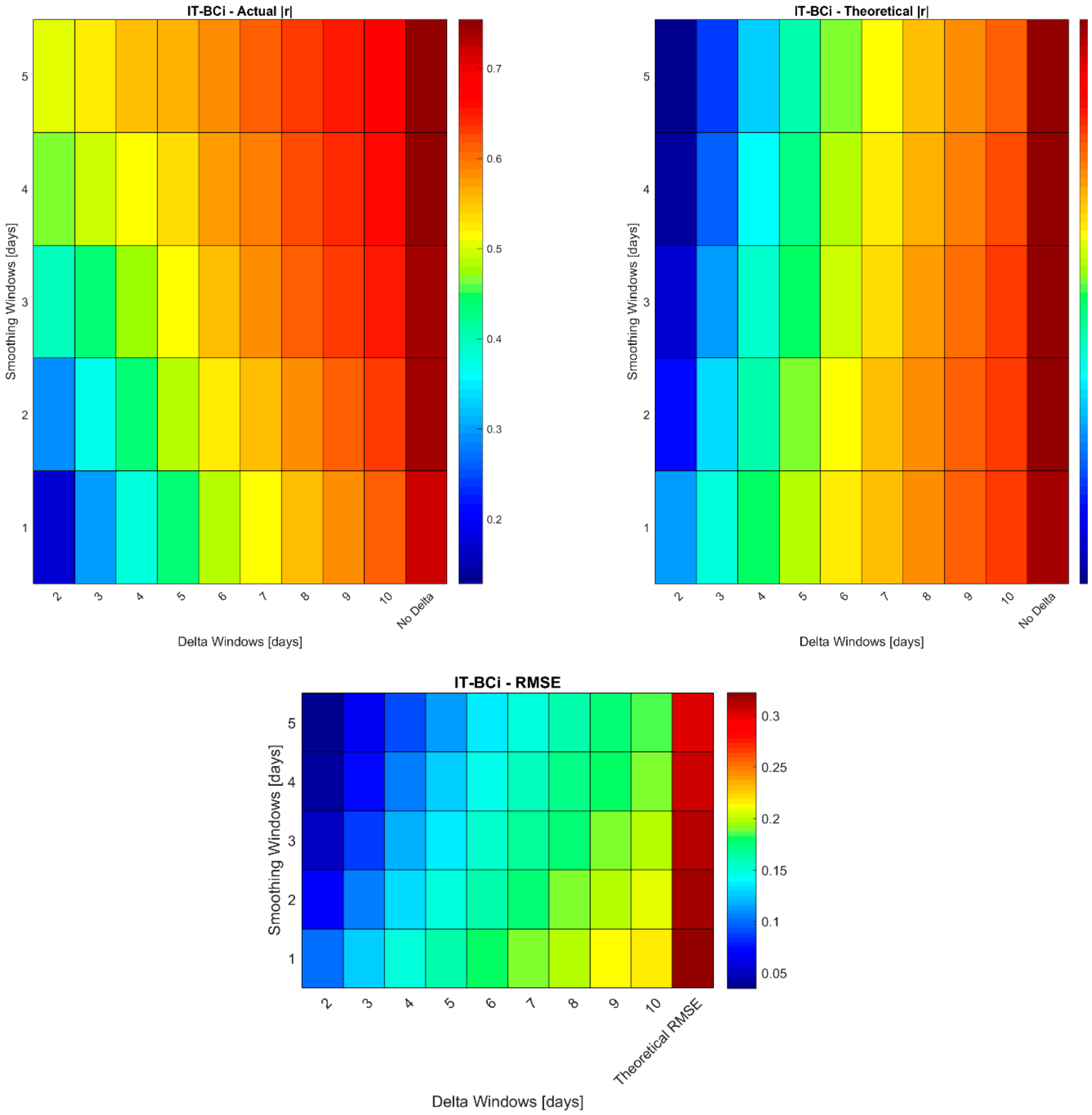

Figure S4. Actual versus theoretical |r| and RMSE performance of the ΔLUE_{Canopy} retrievals at IT-BCi at different levels of smoothing and values of Δ. N = 536.

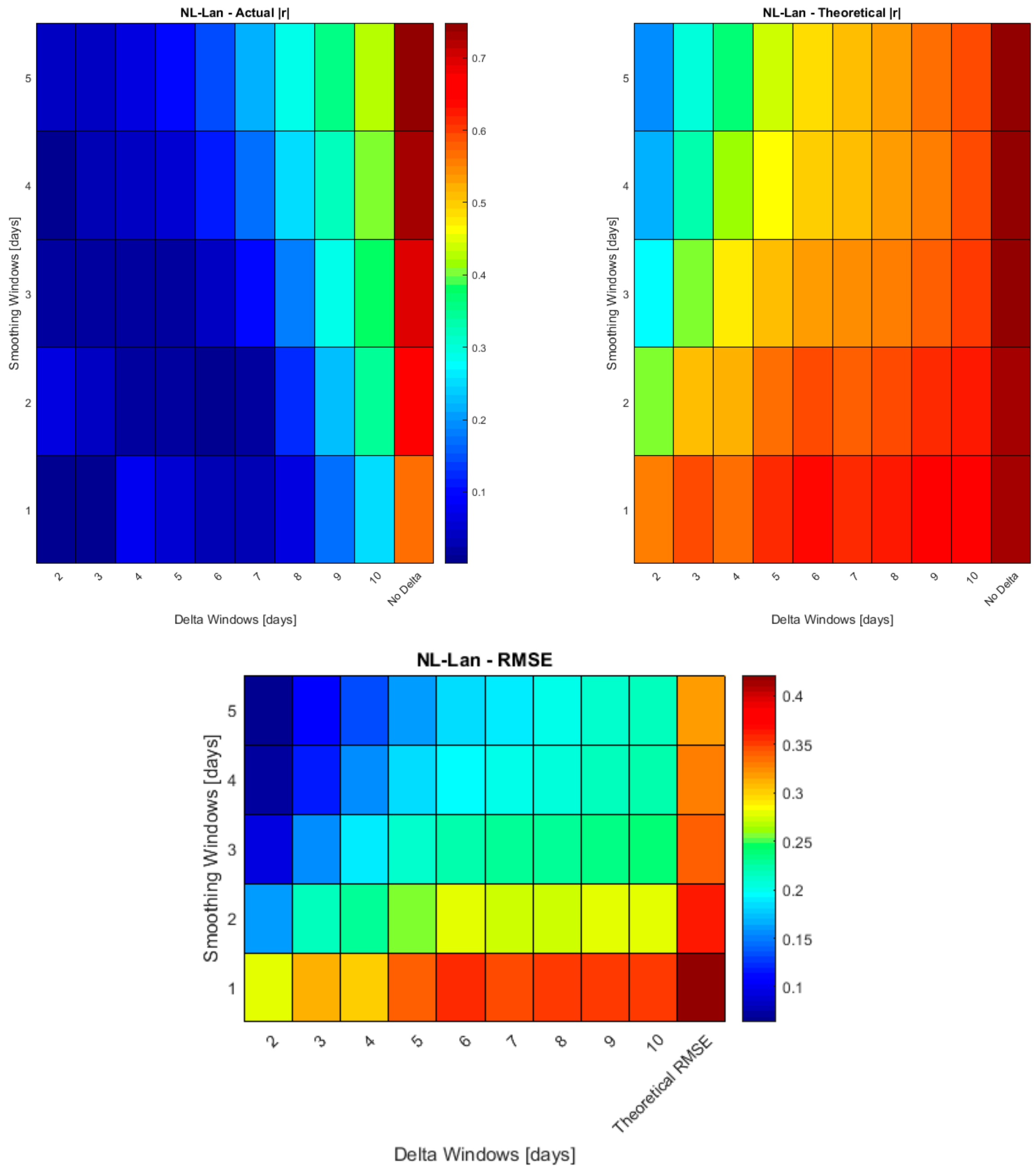

Figure S5. Actual versus theoretical |r| and RMSE performance of the ΔLUE_{Canopy} retrievals at NL-Lan at different levels of smoothing and values of Δ. N = 150.

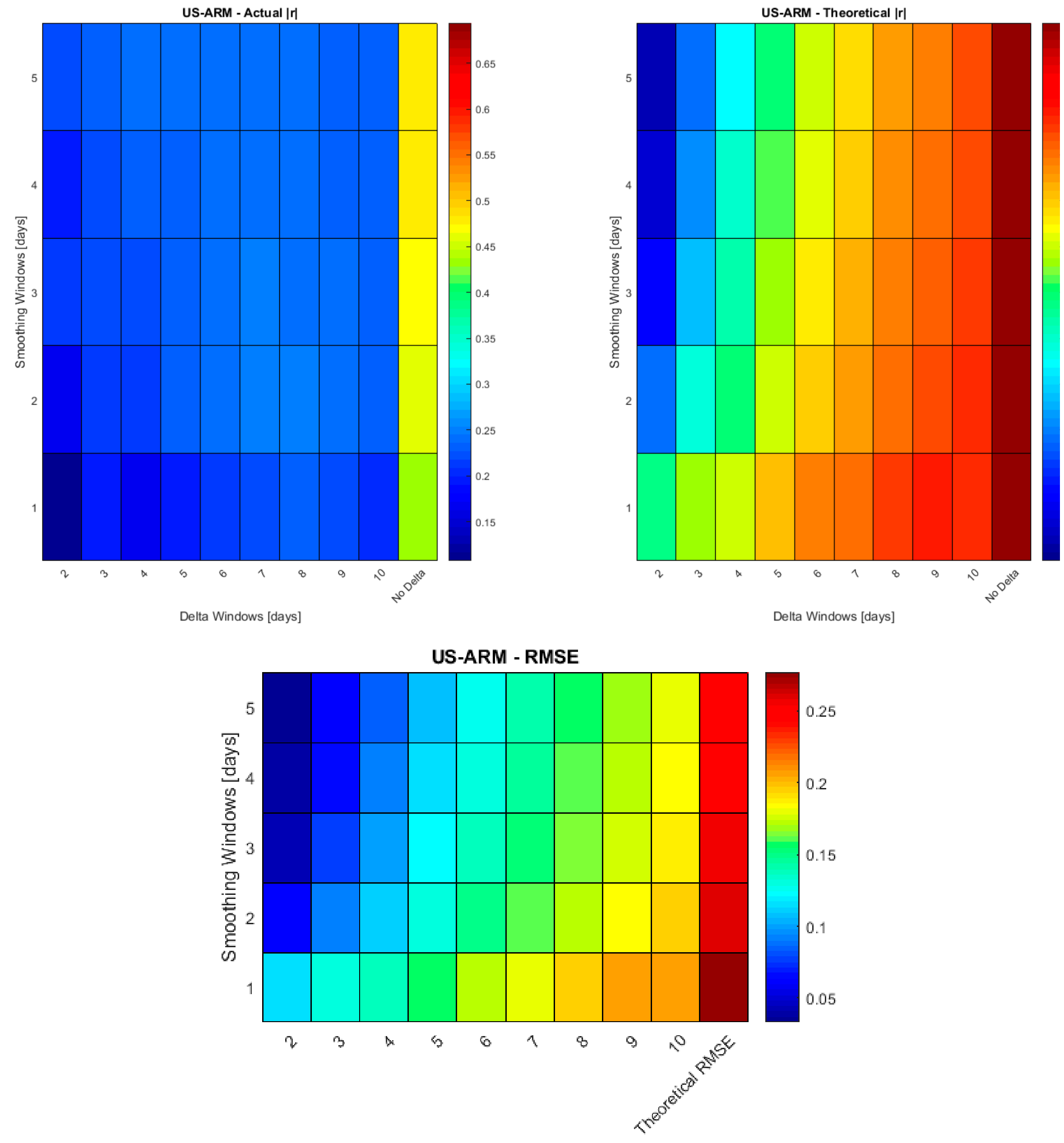

Figure S6. Actual versus theoretical |r| and RMSE performance of the ΔLUE_{Canopy} retrievals at US-ARM at different levels of smoothing and values of Δ. N = 135.

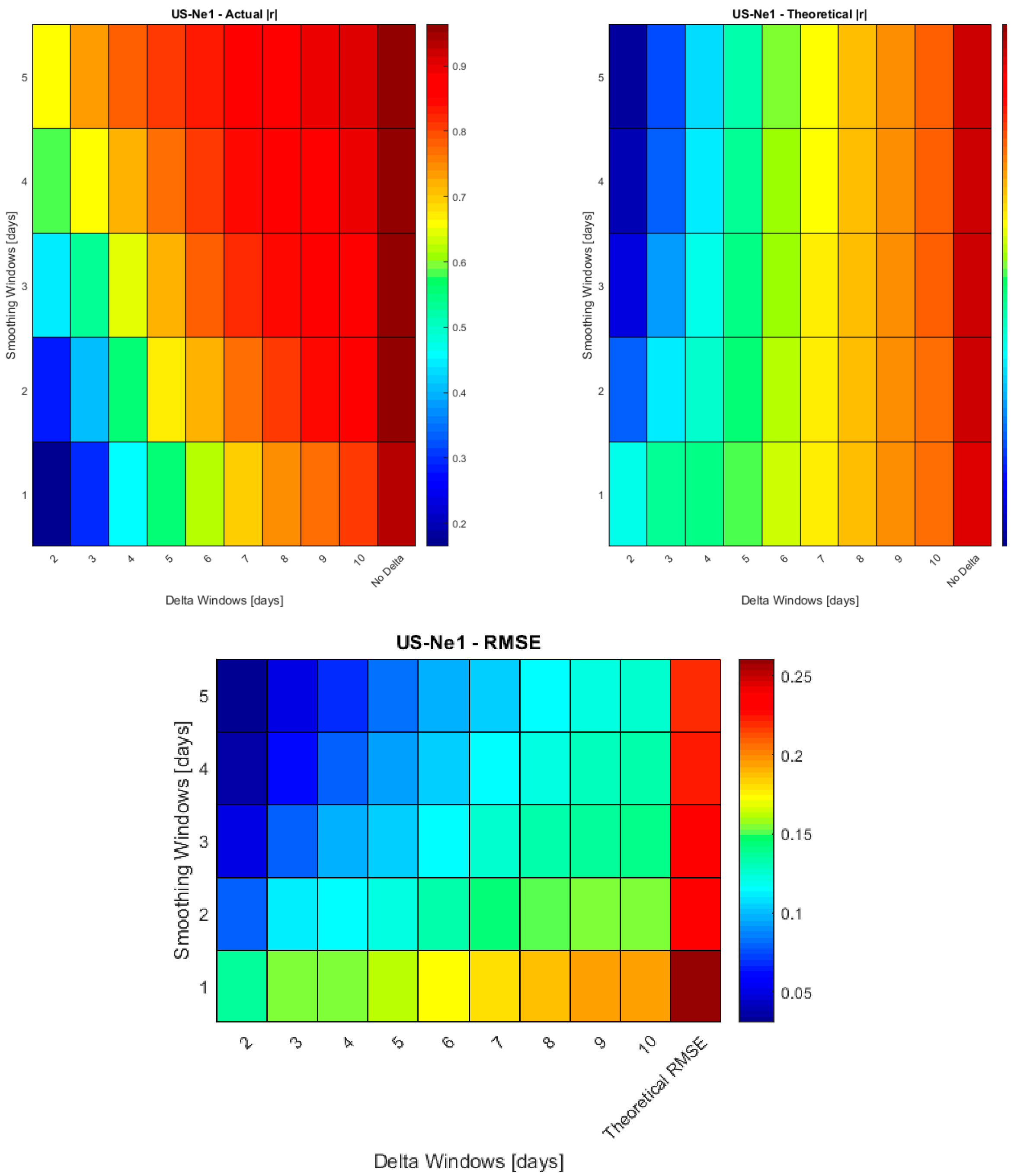

Figure S7. Actual versus theoretical |r| and RMSE performance of the ΔLUE_{Canopy} retrievals at US-Ne1 at different levels of smoothing and values of Δ. N = 1427.

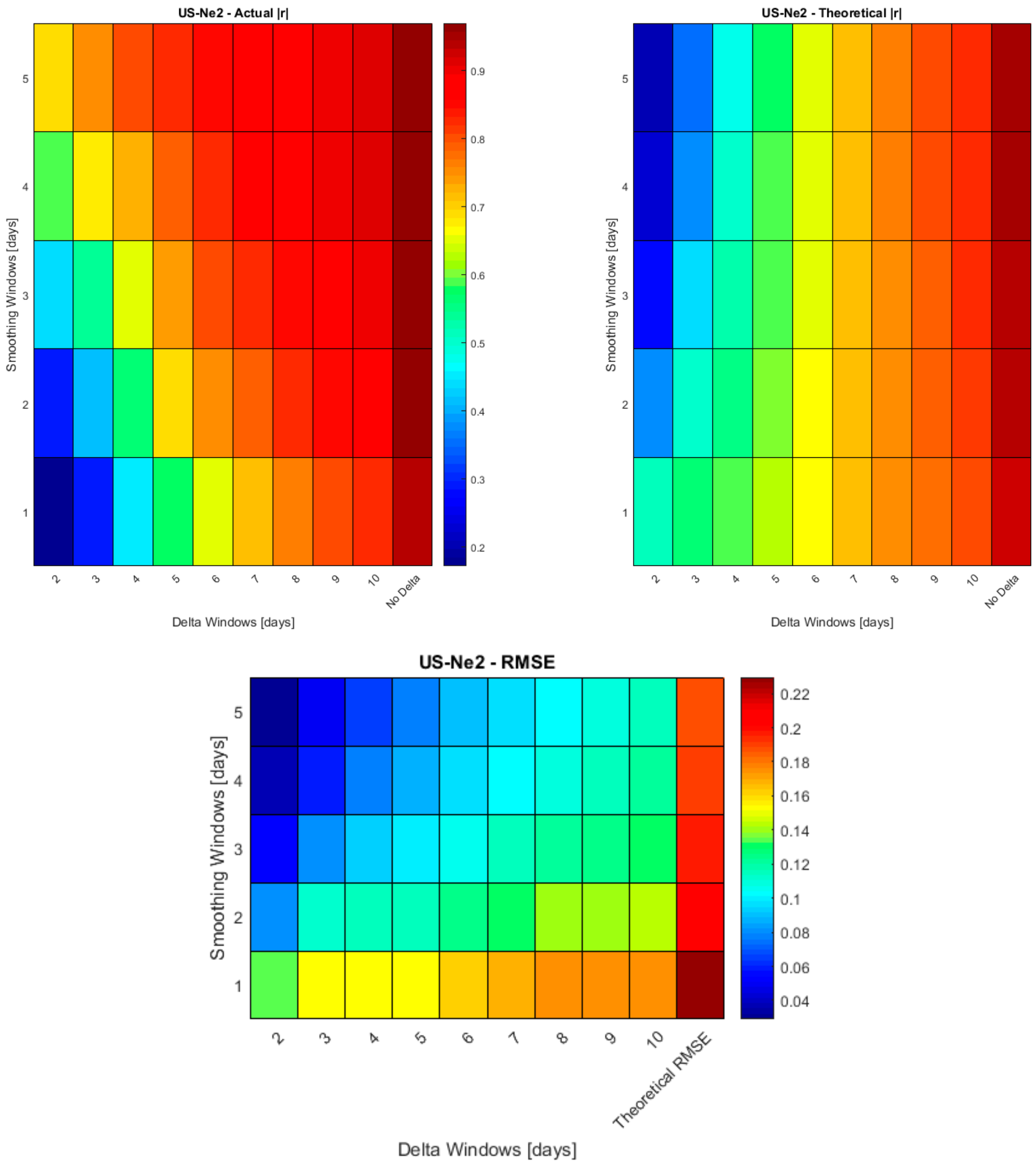

Figure S8. Actual versus theoretical |r| and RMSE performance of the ΔLUE_{Canopy} retrievals at US-Ne2 at different levels of smoothing and values of Δ. N = 799.

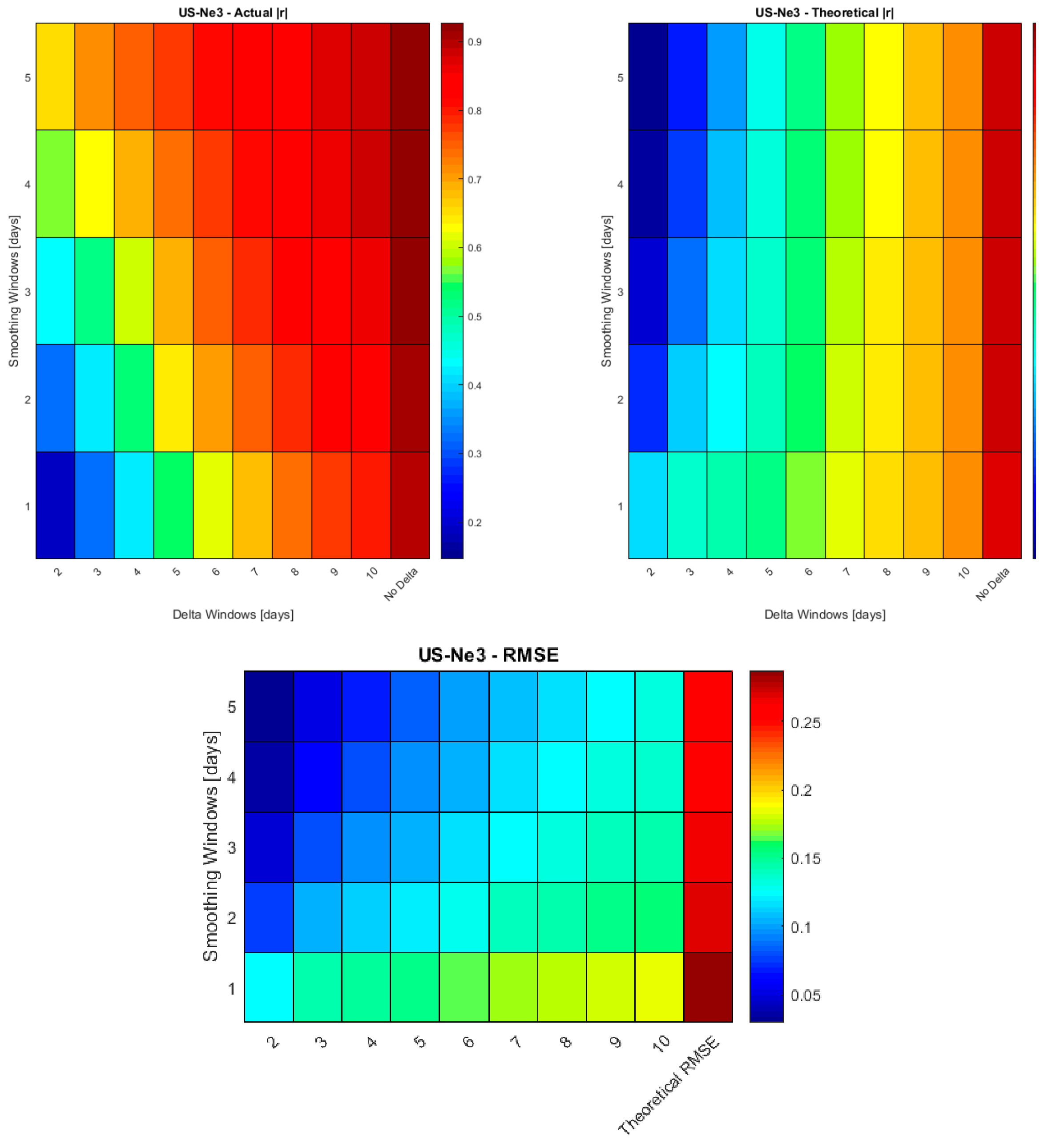

Figure S9. Actual versus theoretical |r| and RMSE performance of the ΔLUE_{Canopy} retrievals at US-Ne3 at different levels of smoothing and values of Δ. N = 786.

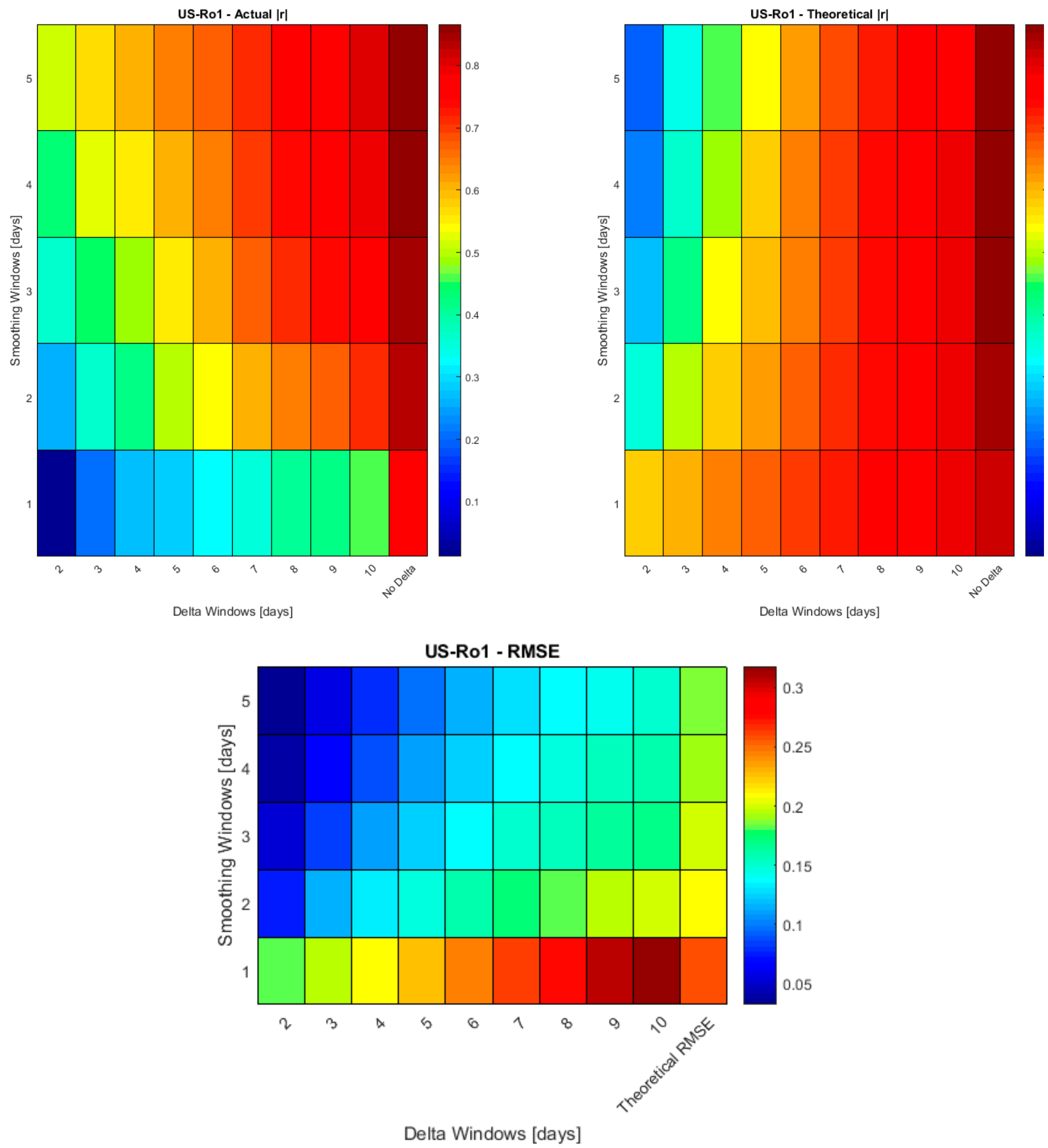

Figure S10. Actual versus theoretical |r| and RMSE performance of the ΔLUE_{Canopy} retrievals at US-Ro1 at different levels of smoothing and values of Δ. N = 263.

© 2019 by the authors; licensee MDPI, Basel, Switzerland. This article is an open access article distributed under the terms and conditions of the Creative Commons by Attribution (CC-BY) license (http://creativecommons.org/licenses/by/4.0/).
